# Self-Organization of Dynein Motors Generates Meiotic Nuclear Oscillations

**DOI:** 10.1371/journal.pbio.1000087

**Published:** 2009-04-21

**Authors:** Sven K Vogel, Nenad Pavin, Nicola Maghelli, Frank Jülicher, Iva M Tolić-Nørrelykke

**Affiliations:** 1 Max Planck Institute of Molecular Cell Biology and Genetics, Dresden, Germany; 2 Max Planck Institute for the Physics of Complex Systems, Dresden, Germany; 3 Department of Physics, Faculty of Science, University of Zagreb, Zagreb, Croatia; Adolf-Butenandt-Institut, Germany

## Abstract

Meiotic nuclear oscillations in the fission yeast Schizosaccharomyces pombe are crucial for proper chromosome pairing and recombination. We report a mechanism of these oscillations on the basis of collective behavior of dynein motors linking the cell cortex and dynamic microtubules that extend from the spindle pole body in opposite directions. By combining quantitative live cell imaging and laser ablation with a theoretical description, we show that dynein dynamically redistributes in the cell in response to load forces, resulting in more dynein attached to the leading than to the trailing microtubules. The redistribution of motors introduces an asymmetry of motor forces pulling in opposite directions, leading to the generation of oscillations. Our work provides the first direct in vivo observation of self-organized dynamic dynein distributions, which, owing to the intrinsic motor properties, generate regular large-scale movements in the cell.

## Author Summary

A key aspect of life is sexual reproduction, which involves the mixing of genetic material during meiosis. In fission yeast and other organisms, the successful mixing and recombining of the chromosomes requires concerted movement of the nucleus. This phenomenon is driven by molecular motors that move the nucleus back and forth inside the cell with the aid of microtubules. How motors and microtubules work together to produce these large-scale movements, however, remains a mystery. Here, we show that nuclear oscillation in fission yeast occurs as motor proteins redistribute from microtubules behind the moving nucleus to those in front of the nucleus, generating an asymmetric distribution of motors and, consequently, of pulling force. By combining quantitative live cell imaging and laser ablation with a theoretical model, we find that this dynamic motor redistribution occurs purely as a result of changes in the mechanical strain sensed by the motor proteins. This work therefore demonstrates that spatio-temporal pattern formation within a cell can occur as a result of self-organization driven by mechanical cues, rather than via the more commonly observed mechanisms of conventional molecular signaling or self-organization driven by biochemical reactions and diffusion.

## Abstract 3

Mechanical cues cause molecular motor proteins to dynamically redistribute within the cell, thereby generating asymmetric patterns of forces that lead to large-scale nuclear movements.

## Introduction

At the onset of meiosis in the fission yeast S. pombe, two cells of opposite mating types fuse at their tips forming a banana-shaped zygote. Subsequently, the two nuclei of the parental cells fuse into one, which starts to oscillate from one end of the cell to the other [1]. These oscillations have a period of about 10 min and last for several hours [1]. The oscillations are crucial for proper chromosome pairing, recombination, and spore viability [2,3]. Similar chromosome movements have been observed in meiotic prophase in a variety of model organisms, from budding yeast to mouse [4,5], and the role of these movements in chromosome pairing and recombination has been demonstrated [6].

The oscillations of the nucleus, also known as the horsetail nuclear movement, follow the oscillatory movement of the spindle pole body (SPB, a centrosome equivalent in yeast) [1]. The movement depends on microtubules [7] and the cortically anchored minus end-directed motor protein dynein [2]. The microtubules extend from the SPB in opposite directions, with the minus ends at the SPB and the plus ends pointing towards the cell periphery [8], while dynein motors accumulate on the SPB and microtubules [2]. Although the key proteins involved in the oscillations have been identified and localized, the underlying physical mechanism is unknown.

By a combination of experiments and theory, we identified the key mechanisms generating the oscillations. Using laser ablation of microtubules, we directly show that oscillations are driven by pulling forces. We visualized and quantified the dynamic distributions of dynein in the cell. We observed an asymmetry in the number of dynein motors attached to oppositely oriented microtubules in response to the microtubule velocity. Using the known force-velocity relationship for dynein motors [9], we interpret this finding as evidence for load-dependent detachment of motors. In response to load forces, dynein motors detach from the trailing microtubules. In particular, the motors detach first from the cell cortex, and then from the microtubule. After redistributing via the cytoplasm, the motors attach along the leading microtubules, thereby producing the force asymmetry necessary for the oscillations. In brief, we propose a novel mechanism for nuclear oscillations based on mechanical regulation of an asymmetric distribution of motors that self-organizes dynamically in the cell.

## Results

### Pulling Forces are Exerted Along the Microtubules

**Figure 1:**
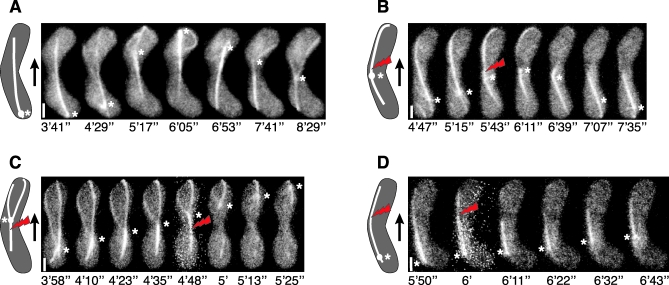
SPB Movement is Driven by Pulling Via the Leading Microtubules (A–D) SPB oscillations in cells expressing Sid4-GFP (SPB marker, indicated by asterisks) and GFP-alpha2-tubulin. Each panel shows selected images from a time-lapse sequence of a cell, together with a scheme of the SPB and microtubules at the time of laser ablation. The red bolt indicates the position of ablation; the black arrows indicate the direction of SPB movement. (SPB position as a function of time for Figure 1B–1D are shown in Figure 6D–6F). (A) Control cell without laser ablation. (B) Laser ablation of the leading microtubules induced a change of direction of the SPB movement (*n* = 19 out of 21 cells; in the remaining two cells another microtubule pulled the SPB in the same direction). Laser ablation of the (C) trailing microtubules (*n* = 19/19), or (D) the tip of the leading microtubules (*n* = 10/10) did not affect the SPB movement. Scale bars, 2 μm.

The nuclear oscillations in S. pombe are lead by the motion of the SPB [1]. This movement depends on microtubules [7], which extend from the SPB towards the two ends of the cell (Figure 1A). The microtubule minus ends are at the SPB, whereas the plus ends point towards the cell periphery [8]. We refer to the microtubules extending from the SPB in the direction of the SPB movement and in the opposite direction as the leading and trailing microtubules, respectively. The leading microtubules are found in close proximity to the cell cortex (Video S1) [8]. Typically, only parts of the length of the trailing microtubules are close to the cortex. Microtubule catastrophe, i.e., the transition from growth to shrinkage, occurs predominantly when the microtubule tip is at the cell end [8]. As the SPB moves, the leading microtubules typically shrink, while the trailing ones grow [8].

**Figure 2:**
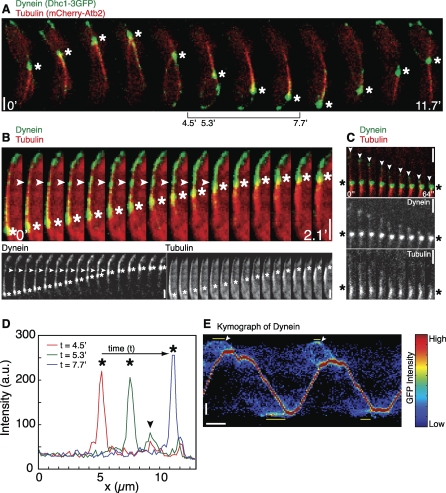
The Dynamics of Dynein Distributions (A) A time-lapse sequence of a cell expressing Dhc1p-3GFP (dynein, green) and mCherry-alpha2-tubulin (red) during one oscillation period. Dynein signal is stronger on the leading than on the trailing microtubules. Time points in minutes corresponding to the intensity profiles in (D) are marked below the image sequence. (B) Time-lapse images of a region in the cell, showing that dynein is distributed in a spotted pattern along the leading microtubules. The spots are stationary with respect to the cell cortex (arrowheads). Lower panels show GFP and mCherry channels separately. (C) Time-lapse images of a region in the cell, showing that when leading microtubules detach from the cortex, dynein also detaches from the cortex and remain on the plus ends of the depolymerizing microtubules (arrowheads). Lower panels show GFP and mCherry channels separately. (D) Dynein intensity in the cell from (A) is shown for the three time points marked below (A). The intensities were calculated as maximum projection along the short axis of the image showing the sum-intensity z-projection of the GFP (dynein) channel, thus the *x*-axis represents the long axis of the cell. The signal of the stationary dynein spot (arrowhead) increases as the SPB (asterisk) approaches it (compare the red and the green curve). The dynein spot is removed from the cortex when the SPB passes over its location (compare the blue curve with the previous two). The arrow shows the movement of the SPB. (E) Kymograph of the GFP channel showing that dynein, which is stationary with respect to the cell cortex (yellow horizontal bars), occasionally detaches (arrowheads) and moves towards the SPB (red trace coming from a strong GFP signal). The white horizontal scale bar represents 2 min. Asterisks in all panels indicate the position of the SPB and the vertical scale bars represent 2 μm.

**Figure 3:**
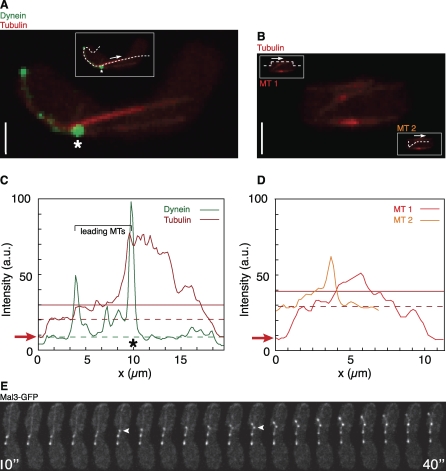
Dynein Resides along the Lateral Sides of Microtubules in Addition to the Plus Ends (A) and (C) image and the corresponding intensity profile of dynein (green) and tubulin (red) for a cell in meiotic prophase. (B) and (D) image and the corresponding intensity profile of tubulin (red) for an interphase cell of the same strain as in (A). The two cells shown in (A) and (B) were in the same field of view, 9 μm apart. (C) and (D) The intensities were measured along the white dashed lines shown in (A) and (B), respectively. The cytoplasmic tubulin signal was estimated as a difference between the cytoplasmic level (dashed red line) and the background level (red arrows). In the interphase cell, the signal of a single microtubule (horizontal solid red line), which is found in the part of the bundle near the cell tip, was ∼50% above the cytoplasmic signal (D). The signal along the leading microtubules in the meiotic cell (horizontal solid red line) was ∼100% above the cytoplasmic signal (C), indicating that there are two leading microtubules in the meiotic cell. The dynein signal (solid green line) shows several peaks along the leading microtubules, above the cytoplasmic level of dynein (dashed green line). This suggests that dynein is present along the microtubules and not only at their ends. (E) A time-lapse sequence of a cell expressing Mal3-GFP (plus end marker) during the SPB movement showing microtubule nucleation events (arrowheads). Nucleation events were identified when the signal was visible also in the previous and the subsequent image. The nucleation rate was 3.5 ± 1.2 min^−1^ (mean ± standard deviation, *n* = 105 events in three cells). Asterisks (A and C) indicate the position of the SPB. Vertical scale bars (A,B,E) represent 2 μm.

Yamamoto et al. [8] proposed that the major force that drives SPB movement is dynein-dependent pulling via the leading microtubules, though pushing by the trailing microtubules may also play a role in the movement [7]. Using laser ablation of specific microtubules [10–13], we perturbed the force balance in the cell to directly test the contribution of pulling and pushing. If the SPB movement is mainly driven by pulling, then ablation of the leading microtubules will disturb the movement, whereas ablation of the trailing microtubules will not affect the movement. To the contrary, if the SPB movement is driven mainly by pushing, then the ablation will have opposite effects. Our experiments showed that after a selective ablation of the leading microtubules the SPB rapidly changed the direction of motion (*n* = 19 out of 21 cells; Figure 1B; Video S2). Ablation of trailing microtubules did not affect the movement (*n* = 19/19; Figure 1C; Video S3). These data provide direct evidence that the SPB movement is driven by pulling via the leading microtubules [8], while the contribution of pushing is negligible.

We next asked whether the pulling force is generated at the interaction site between the leading microtubule tip and the cell end, or largely along the whole length of the leading microtubules [14]. To distinguish between these scenarios, we ablated the leading microtubules ∼4 μm away from the microtubule tip. After the selective ablation of the microtubule tip region, the remaining ∼4-μm-long leading microtubules and the SPB continued to move forward (*n* = 10/10; Figure 1D; Video S4). This observation indicates that the force generated along the lateral microtubule-cortex interactions is large enough to generate the motion of the SPB observed during the oscillations. Additional evidence for force generation along lateral microtubule-cortex interactions is provided by observations of SPB movement in the absence of interactions between the leading microtubules and the cell end in unperturbed cells (Video S1).

### Dynein Redistributes to the Leading Microtubules in Response to the SPB Velocity

The minus end-directed motor cytoplasmic dynein [15] most likely generates the force for the SPB movement. Indeed, dynein heavy chain (Dhc1) deletion results in a lack of oscillations [2], even though meiotic divisions do still occur. Dynein, observed as a Dhc1-green fluorescent protein (GFP), is localized at the SPB and microtubules [2].

In order to understand the interplay between dynein, microtubules, and the SPB movement, we quantified the dynamics of the spatial distribution of dynein during the oscillations. We labeled Dhc1p with triple GFP and tubulin with mCherry (Text S1, II.D). We observed a strong dynein signal on the leading microtubules, and a weak signal on the trailing microtubules (Figure 2A; Video S5). A weak signal on the trailing microtubules was observed irrespective of the proximity of these microtubules to the cortex (Figure S6; Text S1, II.A.1). According to the measured force-velocity curve of dynein [9], a reverse motion, corresponding to the motion of the trailing microtubules, is associated with a high load on the motors. We conclude that the observed comparatively weak dynein signal on trailing microtubules is a consequence of load-dependent detachment of dynein.

### Dynein Resides Along the Lateral Sides of Microtubules in Addition to the Plus Ends

The dynein signal on the leading microtubules was distributed in a spotted pattern (Figure 2B; Video S6). Dynein spots were typically stationary with respect to the cortex while the SPB moved toward them (Figure 2B, 2D, and 2E; nontypical events are presented in Figures 2C, S9, and Text S1, II.A.5). We asked whether the observed spots represent dynein clusters only at the plus ends of individual microtubules or also laterally along the microtubules. The number of dynein spots was measured directly from the images (Figures 3A, 3C, and S8). To determine the number of microtubules, we compared the signal of mCherry-labeled microtubules in cells in meiotic prophase with those in interphase, for which the number of microtubules is known (Figure 3A–3D) [16,17]. Independently, we determined the number of microtubules in meiotic cells as a ratio between the nucleation and the catastrophe rate. We determined the nucleation rate by following growing plus ends using Mal3-GFP (Figure 3E) [18] and used the known catastrophe rate [8]. Both methods yielded two microtubules on the leading side for the example shown in Figure 3A (Text S1, II.A.3). In the same example, the strongest dynein signal along the leading microtubules was found at the point that corresponds to the plus ends of both leading microtubules. There were, however, at least two additional dynein spots along these microtubules. We conclude that dynein resides along the lateral sides of microtubules in addition to the plus ends.

If dynein is distributed along the microtubules, why does it accumulate in spots? Microtubule-bound dynein is linked to the cell cortex by the anchor protein Mcp5/Num1, which is distributed in a spotted pattern at the cortex [19,20]. The spotted pattern of dynein most likely reflects the spotted pattern of the cortical anchor protein.

### Dyneins Linking Microtubules to the Cortex Detach First From the Cortex

Finally, we asked whether the dynein-mediated links between the microtubules and the cell cortex break more easily on the microtubule or the cortex side. We occasionally observed spontaneous breakage of the dynein-mediated links between the plus end of the microtubules and the cortex. In these cases dynein, which was previously stationary with respect to the cortex, detached from the cortex and followed the plus end of the depolymerizing microtubules (Figures 2C, 2E, and S9; Video S7). An alternative mechanism of dynein accumulation at the plus ends of depolymerizing microtubules by plus end-directed motors [21,22] is not likely here (Text S1, II.A.4). We conclude that when the plus end detaches from the cortex, dynein also detaches from the cortex and remains on the microtubule. This suggests that dynein is more tightly bound to the microtubules than to the cortex. Current in vitro studies of motor proteins do not mimic this situation because there the motor tail domain is fixed to a bead or a surface [9,23,24].

### The Minimal Model

In order to identify the key mechanisms necessary to account for the observed SPB movement, we developed a minimal 1-D description on the basis of the above experimental results and the known physical properties of motors. We consider motors that attach to dynamic microtubules and link them to the cortex. The attachment rate depends on microtubule length and motor concentration; the detachment rate is load-dependent. The linked motors generate a force on the microtubules described by a force-velocity relationship.

**Figure 4:**
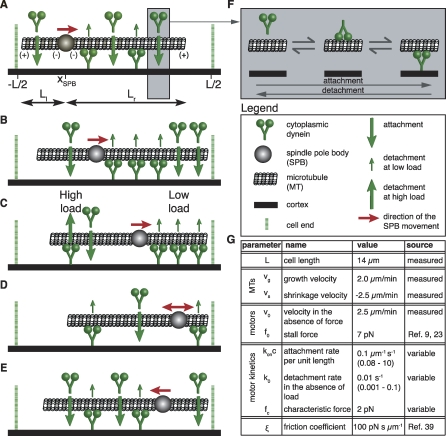
The Minimal Model (A–D) Schematic drawing of events during oscillations. (A) The position of the SPB along the cell longitudinal axis is denoted *x*_SPB_. The two cell ends are located at *x* = −*L*/2 and *x* = *L*/2, respectively, where *L* is the cell length. Two microtubules grow from the SPB. There are more motors attached to the microtubule on the right, thus the SPB moves to the right. As the SPB moves, the motors on the left microtubule are under high load, which stimulates their detachment. The load on the motors on the right microtubule is low. The asymmetry in the number of motors on the two microtubules grows, resulting in a faster SPB movement. (B) The faster movement further increases the asymmetry in the load on the motors, creating a positive feedback between the SPB movement and the number of motors. (C) However, because of the finite size of the cell, the right microtubule shrinks and thus loses motors. (D) When the number of motors on the left and the right microtubule is equal, the SPB does not move. Since the left microtubule is longer than the right one, it accumulates more motors. (E) Thus, the SPB changes direction and the oscillation cycle continues. (F) Steps in the dynein attachment and detachment process; the intermediate step is not included in the model (Text S1, I.F). (G) Parameters of the model. The values in brackets denote the intervals where the behavior of the model does not change. υ_0_ was estimated as the maximum SPB velocity (Figure S7; Text S1, Tables SI–SIII); experimentally measured values of υ_g_, υ_s_, υ_0_ for the strain used in Figures 2, 3A, and 5D–5F are shown in Text S1, Table SII.

The geometry of the minimal model is represented in Figure 4. Two microtubules extend from the SPB in opposite directions. Microtubule dynamics is described by

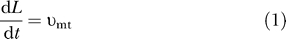
where υ_mt_ = υ_g_ if the microtubule grows and υ_mt_ = υ_s_ when it shrinks. Here, υ_g_ and υ_s_ are the growth and shrinkage velocity, respectively. Equation (1) describes the dynamics of the microtubule growing to the left and to the right if one replaces *L* by *L*_ι_ and *L*_r_, respectively. The transition from growing to shrinking occurs when the plus end of a microtubule reaches the cell end. This assumption is based on experimental observations [8]. Subsequently, the microtubule shrinks until its length vanishes [8]; this is followed by nucleation of a growing microtubule.

The viscous friction force and the forces *F*_ι_ and *F*_r_ acting on the left and the right microtubule, are balanced,

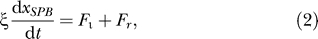
where *x*_SPB_ is the position of the SPB along the long axis of the cell, and ξ the friction coefficient of the system consisting of the nucleus, SPB, and microtubules. The forces *F*_ι_ and *F*_r_ are exerted by attached motors, *F*_ι_ = *N*_ι_ *f*_ι_ and *F*_r_ = *N*_r_ *f*_ r_. Here, *N*_ι_ and *N*_r_ are the total number of motors attached to each microtubule and linked to the cortex, and *f*_ι_ and *f*_r_ the forces generated by a single motor on the left and the right microtubule, respectively. The forces *f*_ι_ and *f*_r_ are described by linear force-velocity relationships, υ *=* υ_0_(1 + *f*_ι_/*f*_0_) and υ *=* υ_0_(*−*1 + *f*_r_/*f*_0_), respectively [9,23,25,26]. The velocity of the motor with respect to the microtubule is υ *=* −υ_SPB_ = −d*x*_SPB_/d*t*, where υ_SPB_ is the SPB velocity. The velocity in the absence of force is denoted υ_0_, and *f*_0_ is the stall force of the motor. The linear densities, *n*_ι_ *= N*_ι_/*L*_ι_ and *n*_r_ *= N*_r_/*L*_r_, of the motors attached to the left and the right microtubule, respectively, obey kinetic equations which describe attachment and detachment of motors. For the right microtubule this equation reads:

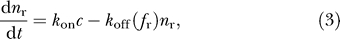
with *n*_r_ *=* 0 at microtubule nucleation. Here, *c* is the cytoplasmic concentration of motors, *k*_on_ characterizes the rate of attachment of motors to microtubules. As in [25,27–30], the load-dependent motor detachment rate, *k*_off_, is described by

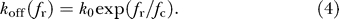
Here, *f*_r_ is the load force acting on individual motors, *k*_0_ is the detachment rate in the absence of a load, and *f*_c_ a characteristic force. The equations that describe the kinetics of motors on the left microtubule are obtained by substituting the subscript *r* by ι in Equations (3) and (4), and changing the sign of the exponent in Equation (4).

Our minimal model differs from models for spindle and chromosome oscillations [25,28–31] in several respects. Here, motors detach from the cortex and redistribute dynamically, whereas in other scenarios they are fixed at the cortex or the chromosomes. Furthermore, in our model the motors coming from the cytoplasm, attach along the length of dynamic microtubules. This implies that a longer microtubule can accumulate more motors than a shorter one, unlike in the other scenarios. Finally, the model discussed here does not include a restoring force such as microtubule pushing [25,28,32].

### Solutions of the Minimal Model

**Figure 5:**
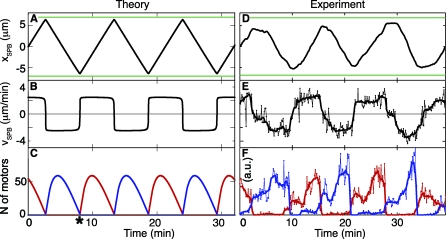
Comparison of Theory and Experiments: Oscillation Pattern and the Number of Motors in Unperturbed Cells (A–C) Numerical results and (D–F) experimental measurements of the time evolution of the SPB position (A, D), SPB velocity (B, E), and the amount of dynein motors on the upper (red) and the lower (blue) microtubules (C, F). (E, F) Thin lines with markers show the original data, while the thick lines show the median in sliding windows of ten data points. (C, F) When the SPB moves upwards (υ > 0), the red curve shows the number of dyneins on the leading microtubule, while the blue curve the number on the trailing microtubule. When the SPB moves downwards (υ < 0), the blue curve corresponds to the leading microtubule, and the red curve to the trailing microtubule. The numerical and the experimental results are plotted with the same scale (A and D, B and E), whereas the scaling factor between (C) and (F) is not known. The different shape of the curves in (C) and (F) is most likely due to the assumption of a uniform distribution of dynein along the microtubule in the model, whereas experiments showed a nonuniform distribution (Figures 2 and 3). The numerical solutions (A–C) can be understood in a simple limit where the SPB moves with the maximal dynein velocity, d*x*_SPB_/d*t* = ±υ_0_ (Text S1, I.C). In this limit, oscillations have an exact triangular waveform. Furthermore, the dynein linear densities on the microtubules obey d*n*/d*t* ≈ *k*_on_ *c* and d*n*/d*t* ≈ 0, giving a parabolic dependence of the total number of dyneins at the leading microtubule on time, and small dynein numbers on the trailing microtubule, respectively (C). Asterisk (C) marks an example of a change of direction of the SPB motion. The results in (D–F) come from a single cell; the averaged results based on 11 cells are shown in Figure S10. SPB velocity as a function of the difference in the number of motors on each side of the SPB is shown in Figure S11.

Independently on initial conditions, numerical solutions of the Equations (1–4) attain limit cycles for typical parameter values (Figure 4G). The resulting oscillations of the SPB position have an almost triangular waveform, with an amplitude slightly smaller than *L*/2 (Figure 5A). This is reflected in the square waveform of the SPB velocity (Figure 5B). The total number of dyneins linking the microtubules to the cortex depends on the SPB velocity: when the direction of motion of the SPB changes (e.g., the time marked by the asterisk in Figure 5C), the number of dyneins attached to the new leading microtubule increases. Simultaneously, the number of dyneins at the now trailing microtubule decreases (Figure 5C). The rapid detachment of dyneins from the trailing microtubule results from the high load force experienced by dyneins that oppose the SPB movement. Compared to dyneins on the trailing microtubule, dyneins on the leading microtubule experience lower load force and thus have a lower detachment rate, which allows for their accumulation. In brief, the oscillations are driven by the changes in the load-dependent detachment rate, while the attachment process does not depend on whether the microtubule is leading or trailing.

The triangular waveform and the small number of dyneins on the trailing microtubule are observed in our minimal model in the regime of a strong dependence of the detachment rate on load (*f*_c_ ≲ 4 pN). In the regime of a weak load-dependence (*f*_c_ ≳ 10 pN), oscillations still occur but the waveform is close to sinusoidal and dynein accumulates on the trailing microtubule (Figures S2 and S3; Text S1, I.B). In each of the two regimes, the waveform and the dynein distribution is robust for a large range of parameters of the minimal model (Figure S1; Text S1, I.B).

### Generalizations of the Minimal Model

Our experiments (Figure 2C) suggest that the dyneins linking the microtubule to the cortex detach in two steps. First, they detach from the cortex but remain on the microtubule. In the second step, they detach from the microtubule and are released into the cytoplasm. In addition, we did not observe motors attached to the cortex in regions devoid of microtubules (Figure 2A–2C).

We have generalized our minimal model to take into account this two-step process (Text S1, I.F). In this model, we distinguish two populations of motors on a microtubule: those attached only to the microtubule, and those linking the microtubule to the cortex. The behavior of the generalized model is similar to that of the minimal model (Figure S5). For rates of detachment of motors from microtubules, similar to those measured in vitro [33], the generalized model reproduces the small number of motors on the trailing microtubule as observed in our experiments (Figures 2A, 2B, and S5; Text S1, I.F). The reason for the small number of dyneins on the trailing microtubules is that after a load-induced detachment from the cortex, dynein is released from the microtubule into the cytoplasm quickly compared to the period of the oscillations.

In addition, we generalized the minimal model to consider multiple microtubules at each side of the SPB, as observed in experiments [8]. The behavior of the generalized model is similar to that of the minimal model in the strong load-dependence regime. In the weak load-dependence regime, on the other hand, oscillations do not exist for multiple microtubules (Figure S4; Text S1, I.D, I.E).

### Experimental Tests of the Minimal Model

In order to determine the role of load-dependence for the observed oscillations, we compared the measured waveforms of the SPB position and velocity with those from theory, obtained in the regimes with strong and weak load-dependence. The measured waveforms of the SPB position and velocity are close to triangular and square waveforms, respectively (Figure 5D and 5E). They match the theoretical prediction in the strong load-dependence regime (Figure 5A and 5B). Consequently, in this regime the theory predicts a low number of motors on the trailing microtubule, owing to a load-dependent detachment rate and redistribution of motors (Figure 5C). Indeed, experiments independently showed the absence of dynein on the trailing microtubules (Figure 5F). Moreover, the model accounts for the experimentally observed end-to-end SPB oscillations (Figure 5A and 5D) if the microtubule shrinkage velocity (υ_s_) is comparable to the maximal motor velocity (υ_0_).

**Figure 6:**
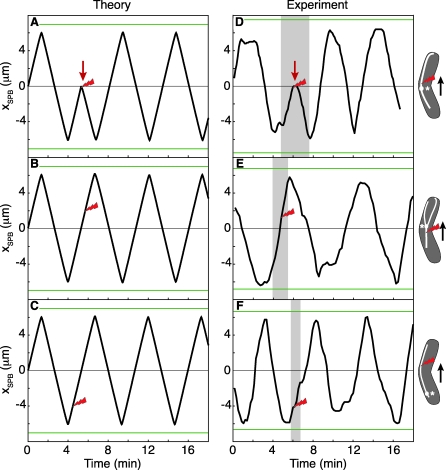
Comparison of Theory and Experiments: Oscillation Pattern after Microtubule Cutting (A–C) Numerical results and (D–F) experimental measurements of the SPB position as a function of time. The red bolt signs mark the time and the SPB position at the moment of microtubule cutting. Schemes show the SPB and the microtubules, as well as the site of laser ablation (bolt signs); the black arrows indicate the direction of SPB movement. Laser ablation of the leading microtubules induced a change of direction of the SPB movement (red arrows in [A and D]). In experiments, the change occurred within 25 ± 13 s after the ablation (mean ± standard deviation, *n* = 12). Laser ablation of the trailing microtubules (B, E) or the tip of the leading microtubules (C, F) did not perturb the SPB movement. The parameters in (A–C) are as in Figure 4, except υ_0_ = 5 μm/min, υ_s_ = −5 μm/min, and υ_g_ = 3 μm/min corresponding to the strain SV28xSV31 (Text S1, Table SIII). The gray rectangles in (D–F) mark the time interval of the images shown in Figures 1B–1D; the green lines mark the cell ends.

The SPB movement can be perturbed by laser-cutting of microtubules (Figure 1). Laser-cutting of the leading microtubules close to the SPB resulted in a rapid reversal of the SPB movement (Figures 1B and 6D). We used the conclusion from this experiment, that SPB movement is driven by microtubule pulling, to build the model. The same experiment, however, provides additional information such as the rapid reversal of the SPB movement, which allows for testing of the model. We simulated the cutting experiment in the model by setting the length of the leading microtubule to zero instantaneously when the SPB was close to the center. This mimicked the experimental situation after laser cutting, where the length of the leading microtubules was below the resolution of the microscope. After the disappearance of the leading microtubule in the simulations, the SPB quickly changed the direction of motion (Figure 6A), in agreement with the experiments (Figure 6D).

For completeness, we tested whether the model is able to reproduce the remaining two cutting experiments (Figure 1C and 1D). Laser-cutting of the trailing microtubules close to the SPB, or cutting of the leading microtubules ∼4 μm away from the SPB, did not perturb the SPB movement (Figures 1C, 1D, 6E, and 6F). Mimicking the laser-cutting of the trailing microtubules in the model by instantaneously setting the length of the trailing microtubule to zero did not affect the SPB movement (Figure 6B). Similarly, the SPB movement remained unchanged when the length of the leading microtubule was instantaneously decreased to 4 μm, after which the microtubule resumed growth (Figure 6C). Thus, the behavior of the model is consistent with microtubule cutting experiments.

The minimal model does not account for details such as the frequent SPB pausing at the cell end (denoted Phase II in [8]; see also Figure 5D and Video S1), and dynein accumulation in dots and at the SPB (Figures 2 and 3) [8].

## Discussion

### Redistribution of Dynein Motors

By using fluorescent live cell imaging, we showed that dynein motors dynamically redistribute in response to microtubule velocity during the nuclear oscillations. Quantifying the signal of dynein motors on the oppositely oriented microtubules revealed that the asymmetry of the dynein motor distribution increases as the SPB moves from one end of the cell to the other.

To describe this asymmetry, we define two populations of dynein motors that are bound to oppositely oriented microtubules: the motors attached to the leading microtubules walk toward the minus end of the microtubules whereas those attached to the trailing microtubules are forced, by the microtubule movement, to move towards the plus end of the microtubules. Therefore, using the known force-velocity relationship of dynein motors [9], we conclude that the dynein population forced to move towards the plus end of the microtubules is under higher load force.

What may be the consequence of high load force in the system? In vitro studies have shown that motors detach from microtubules in response to load forces [24], which is a general property of many motor proteins [34]. This process, known as load-dependent detachment, has been suggested to play a crucial role in spindle and chromosome oscillations [25,28–31]. In our system, the motors on the trailing microtubules are under high load. We interpret the observed small number of motors on the trailing compared to the leading microtubules as a consequence of load-dependent detachment.

We conclude that dynein motors detach and redistribute in response to load forces. This redistribution generates the force asymmetry necessary for the oscillations.

### Microtubule Length-Dependent Motor Attachment

Dynein motors from the cytoplasm can attach along the microtubules and the number of attachment sites depends on the length of the microtubules. Thus we refer to this process as length-dependent attachment. Attachment of dynein along the length of the microtubules is confirmed directly by imaging dynein motors (Figure 3) and indirectly by laser ablation (Figure 1B and 1D).

We suggest that length-dependent attachment of dynein is necessary for the change of direction of the SPB movement. When the movement of the SPB stops near a cell end, the microtubules extending towards the opposite cell end are longer than those pointing to the proximal cell end. Because of the length-dependent attachment of dynein, more motors attach to the longer microtubules. Simultaneously, the load experienced by motors on all microtubules is similar, and therefore the motor detachment rate is also similar. Consequently, longer microtubules accumulate more motors and the movement starts in the direction of the longer microtubules. Once the movement has started, the motors on the longer microtubules experience lower load forces, the motor detachment rate thus decreases and the motors accumulate on the longer microtubules, thereby the change of direction is completed.

During the SPB movement, the leading microtubules shrink while the trailing ones grow. When the leading microtubules become shorter than the trailing ones, more motors attach to the trailing microtubules because of the length-dependent attachment. However, more motors detach from the trailing than from the leading microtubules owing to the load-dependent detachment. If the load-dependent detachment dominates over the length-dependent attachment, shorter microtubules can lead the movement. Pulling by shorter leading microtubules was observed experimentally (Figure 2A) and is a prerequisite for the oscillations. This mechanism differs from those where the pulling force depends on the length of the microtubule [14,35].

### Dynein Motors Detach from the Cell Cortex

We show that dynein detaches both from the cortex and from the microtubule, but not simultaneously. On the basis of the observation that dynein remains on the plus end of the microtubule after the release of the plus end from the cortex, we conclude that dynein first detaches from the cortex and then from the microtubule (Figure 2C). This finding differs from situations where force generators detach from microtubule ends but remain localized on the cell cortex [25,28] or chromosomes [30]. The detachment from the cortex observed here allows for dynein to diffuse in the cell and thus is essential to generate the observed periodic redistribution of dynein that accompanies nuclear oscillations.

### Conclusion

Our work emphasizes that self-organized collective behavior of motors acting on dynamic microtubules can account for large-scale movements of the nucleus during meiotic prophase, without a requirement for additional spatio-temporal regulation of motor activity. We demonstrate that load-dependent detachment of motors occurs in vivo, and that it introduces the dynamic instability necessary for the oscillations. Dynein motors, by responding to load forces, dynamically redistribute in the cell and attach to the microtubules extending in front of the moving SPB, thereby generating oscillations. The dynamic redistribution of motors provides a novel centering mechanism and no other centering forces are required.

Dynamic protein distributions in cells are traditionally interpreted in terms of reaction-diffusion mechanisms [36]. Here, we have shown that load-dependent collective action on motor proteins can generate spatio-temporal patterns by a very different mechanism.

## Materials and Methods

Cell preparation and induction of meiosis was performed following standard procedures (Text S1). Strains and gene tagging (Text S1, Table SIV) were obtained using a PCR gene-targeting method [37] and/or standard genetic methods [38]. Live cell imaging was performed on a spinning disk system, laser scanning confocal system, or a two-photon setup. Laser ablation was performed on a two-photon setup [12]. Cutting of microtubules, performed while continuously scanning a stack, was achieved by focusing the laser over a user-defined point and increasing the average power to 70 mW on the sample. The exposure time, controlled by the computer, was 150 ms. Additionally, laser ablation was performed on a confocal microscope [13]. Images were analyzed using Image J (NIH) and Matlab (MathWorks). A detailed description of the experimental methods and data analysis can be found in Text S1.

## Supporting Information

Figure S1 Model Behavior for Different Values of Parameters (A, D, G) Position of the SPB; (B, E, H) velocity of the SPB; (C, F, I) the number of motors attached to the left microtubules (blue) and the right microtubules (red) as a function of time, for *k*_0_ = 0.001 s^−1^, *k*_0_ = 0.1 s^−1^, and *υ*_s_ = 5 μm/min. In order to obtain roughly the same number of attached motors, we chose *k*_on_ *c* = 0.02 μm^−1^s^−1^ in (A–C), *k*_on_ *c* = 0.5 μm^−1^s^−1^ in (D–F), and *k*_on_ *c* = 0.1 μm^−1^s^−1^ in (G–I). In each case, all the remaining parameters are as in Figure 4G.
(659 KB EPS)

Figure S2Bifurcation Diagram for the Minimal ModelThe amplitude, 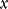
_SPB_ *,* of the oscillations is plotted as a function of *k*_on_ *c* for four different values of *f*_c_. The hollow circle marks the position in the parameter space corresponding to Figures 4 and 5. For *f*_c_ = 1 pN, hysteresis occurs at *k*_on_ *c* ≈ 0.035 − 0.073 μm^−1^s^−1^.
(318 KB EPS)

Figure S3 Behavior of the Model in Strong Load-Dependence Regime (A–C) *f*_c_ = 4 pN, and weak load-dependence regime (D − F) *f*_c_ = 10 pN (A, D) Position and (B, E) velocity of the SPB; (C, F) number of motors attached to the left microtubules (blue) and the right microtubules (red) as a function of time. In each case, all the remaining parameters are as in Figure 4G.
(723 KB EPS)

Figure S4 Influence of the Load-Dependence on the Behavior of the Model with a Single and with Multiple Microtubules (A, D, G, J) Position of the SPB; (B, E, H, K) velocity of the SPB; (C, F, I, L) the number of motors attached to the left microtubules (blue) and the right microtubules (red) as a function of time. The parameters for the model with single microtubules are as in Figure 4G. For the model with multiple microtubules, *T*_nucl_ = 40 s, *k*_on_ *c* = 0.01 μm^−1^s^−1^, whereas the remaining parameters are as in Figure 4G. In the model with multiple microtubules, the average number of microtubules on each side is 16.
(902 KB EPS)

Figure S5Two-Step Attachment and Detachment of Dynein(A, D, G) Position of the SPB; (B, E, H) velocity of the SPB; (C, F, I) the total number of motors attached to the left microtubules (*n*_ι_ *L*_ι_, full blue line) and the number of motors attached both to the cortex and to the left microtubules (*n*_ι*,*MTC_ *L*_ι_, dotted blue line); the total number of motors attached to the right microtubules (*n*_r_ *L*_r_, full red line) and the number of motors attached both to the cortex and to the right microtubules (*n*_r,MTC_ *L*_r_, dotted red line). The results are shown for 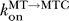 = 1.0 s^−1^ and for three values of 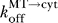 . All the remaining parameters are as in Figure 4G.
(917 KB EPS)

Figure S6 A Time-Lapse Sequence of a Cell Expressing Dhc1p-3GFP (Dynein, Green) and mCherry-alpha2-tubulin (Red) During the Oscillations The asymmetric dynein distribution did not change when the trailing microtubules were in close contact with the cell cortex. The arrowheads and the bent line indicate the close contact between the trailing MTs and the cell cortex. The arrow points to trailing MTs interacting with the cell end. The position of the SPB is marked with asterisks. The scale bar represents 2 μm.
(700 KB EPS)

Figure S7 SPB Position as a Function of Time for the Strain (A) KT233 (Sid4-GFP), (B) SV28xSV31 (Sid4-GFP, GFP-Atb2), (C) JW785 (GFP-Dhc1), and (D) SV81 (Dhc1-3GFP, mCherry-Atb2) The vertical lines mark the fitting range. Each color represents a single cell. For details see Text S1, IIA6.
(5.84 MB EPS)

Figure S8 Dynein Resides along the Lateral Sides of Microtubules in Addition to the Plus End (A) and (B) show a zygote expressing Dhc1p-3GFP (dynein, green) and mCherry-alpha2-tubulin (red) at two different time points during the oscillations, together with interphase cells in the same field of view. Note the high amount of dynein distributed along the leading microtubules.
(C) shows another zygote, also expressing Dhc1p-3GFP (dynein, green) and mCherry-alpha2-tubulin (red).
(D) shows the intensity line profiles of dynein (green) and tubulin (red) measured along the white dashed line in (C). The estimated cytoplasmic intensity of dynein and tubulin are indicated by a dashed green and red line, respectively. The tubulin signal along the leading microtubules is constant (noisy solid red line shows the data, while the horizontal solid red line is the estimated signal corresponding to the leading microtubules), whereas the dynein signal (solid green line) shows several peaks along the same path (D), demonstrating that dynein is present along the microtubules and not only at their plus ends. Asterisks mark the SPB, vertical scale bars represent 2 μm.
(3.52 MB EPS)

Figure S9 The Image Sequences Are Taken from Video S6
(A) frames 5–32 with a time interval of 16 s between the images; (B) frames 121–131 with a time interval of 8 s between the images. The zygote expresses Dhc1p-3GFP (dynein, green) and mCherry-alpha2-tubulin (red). (A) shows a breakage of the leading microtubules. The first arrow marks the breakage of the microtubule, while the following arrows point to the depolymerizing fragment. Note that dynein stays attached to the depolymerizing microtubule fragment.
(B) shows detachment of a leading microtubule from the cortex. The arrowheads point to the depolymerizing plus end of the microtubule. Note that dynein stays attached to the microtubule plus end. Asterisks indicate the position of the SPB. Scale bars represent 2 μm.
(867 KB EPS)

Figure S10 Experimental Measurements of the SPB Position (Black), the Number of Motors on the Leading Microtubule (Red), and on the Trailing Microtubule (Blue) The number of motors is in arbitrary units, and was calculated from the dynein intensity. The data shows the mean value of 50 half-periods from 11 cells. The error bars represent standard error of the mean.
(344 KB EPS)

Figure S11 Switch-Like Redistribution of Dynein Experimental measurements (A) and numerical calculation (B) of the velocity of the SPB, υ_SPB_, as a function of the difference in the number of motors, Δ*N*, on either side of the SPB. The number of motors in (A) is in arbitrary units, and was calculated from the dynein intensity. For details see Text S1, IIG5. The points represent the mean ± standard deviation. The total number of data points is 3,000, calculated from 13 cells.
For the numerical calculation (B), the parameter values are as in Figure 4G.
(313 KB EPS)

Text S1 Click here for additional data file. Self-Organization of Dynein Motors Generates Meiotic Nuclear Oscillations
(1.34 MB PDF)

Video S1 SPB Movement Via Microtubules SPB movement in a zygote during meiotic prophase expressing Sid4-GFP (SPB marker) and GFP-alpha2-tubulin. The zygote was obtained by crossing of strains SV28 and SV31. Images were acquired at 4-s intervals. The video is displayed at 10 frames per second (fps). Total time: 14 min.
(660 KB AVI)

Video S2 Laser Ablation of the Leading Microtubules Affects the SPB Movement A meiotic prophase zygote expressing Sid4-GFP (SPB marker) and GFP-alpha2-tubulin where the ablation of the front microtubules leads to a change in the direction of the SPB motion. The red arrowhead marks the ablation position. The movie pauses for 2 s at the ablation time. The zygote was obtained by crossing of strains SV28 and SV31. Images were acquired at 7-s intervals. The video is displayed at 5 fps. Total time: 10 min 15 s. Corresponds to Figure 1B.
(842 KB AVI)

Video S3 Laser Ablation of the Trailing Microtubules Does Not Affect the SPB Movement A meiotic prophase zygote expressing Sid4-GFP (SPB marker) and GFP-alpha2-tubulin where the ablation of the trailing microtubules does not influence the SPB movement. The red arrowhead marks the ablation position. The movie pauses for 2 s at the ablation time. The zygote was obtained by crossing of strains SV28 and SV31. Images were acquired at 12.5-s intervals. The video is displayed at 5 fps. Total time: 14 min 35 s. Corresponds to Figure 1C.
(504 KB AVI)

Video S4 Laser Ablation of the Tip of the Leading Microtubules Does Not Affect the SPB Movement A meiotic prophase zygote expressing Sid4-GFP (SPB marker) and GFP-alpha2-tubulin where the ablation of the tip of the leading microtubules does not influence the SPB movement. The red arrowhead marks the ablation position. The movie pauses for 2 s at the ablation time. The zygote was obtained by crossing of strains SV28 and SV31. Images were acquired at 10.6-s intervals. The video is displayed at 5 fps. Total time: 9 min. Corresponds to Figure 1D.
(560.39 KB AVI)

Video S5 Dynein Signal Is Stronger on the Leading Than on the Trailing Microtubules A meiotic prophase zygote expressing Dhc1-3GFP and mCherry-alpha2-tubulin (strain SV81). Note that the leading microtubules show a strong dynein signal while the dynein signal along the trailing microtubules is weak. Images were acquired at 8-s intervals. The video is displayed at 15 fps. Total time: 64 min 56 s. Corresponds to Figures 2A and S6.
(4.43 MB AVI)

Video S6 Dynein Is Distributed in a Spotted Pattern along the Leading Microtubules A meiotic prophase zygote expressing Dhc1-3GFP and mCherry-alpha2-tubulin (strain SV81). Note that dynein is distributed in a spotted pattern along the leading microtubules. The spotted pattern is stationary with respect to the cortex. Images were acquired at 8-s intervals. The video is displayed at 15 fps. Total time: 17 min 28 s. Corresponds to Figures 2B and S9.
(1.17 MB AVI)

Video S7 Dynein Remains on the End of Depolymerizing Microtubules A meiotic prophase zygote expressing Dhc1-3GFP and mCherry-alpha2-tubulin (strain SV81). Note that when the microtubule detaches from the cortex, dynein remains on the plus-end during microtubule depolymerization. Images were acquired at 8-s intervals. The video is displayed at 10 fps. Total time: 1 min 4 s.
Corresponds to Figure 2C.
(47.32 KB AVI)

## Abbreviations

Dhc1: dynein heavy chain
fps: frames per second
GFP: green fluorescent protein
SPB: spindle pole body
