# Supplementary material for: Self-Organization of Dynein Motors Generates Meiotic Nuclear Oscillations
Source: PLoS Biol. 2009 Apr 21;7(4):e1000087. doi: 10.1371/journal.pbio.1000087 (PMC2671556; doi:10.1371/journal.pbio.1000087)
Supplement: Supplementary file 1 [file pbio.1000087.sd001.pdf]

Supporting Information: Self-organization of Dynein Motors Generates Meiotic Nuclear Oscillations

Sven K. Vogel,[1,*] Nenad Pavin,[2,3,*] Nicola Maghelli,[1] Frank Jülicher,[2] and Iva M. Tolić-Nørrelykke[1]

[1]*Max Planck Institute of Molecular Cell Biology and Genetics, Dresden 01307, Germany*
[2]*Max Planck Institute for the Physics of Complex Systems, Dresden 01187, Germany*
[3]*Department of Physics, Faculty of Science, University of Zagreb, Zagreb 10002, Croatia*

CONTENTS

I. Theory 2
- A. Minimal model 2
- B. The influence of parameter changes on the minimal model 2
- C. Analytical solution of the minimal model 5
- D. Multiple microtubules 5
- E. Sensitivity of the model to load-dependence 6
- F. Two-step attachment and detachment of dynein 6
- G. Nonlinear force-velocity relationship 7
- H. Differences between our model and other models involving microtubule pulling forces from the cortex 8

II. Experiments 8
- A. Supplementary experimental results 8
  1. The asymmetry of the dynein distribution was observed also when the trailing microtubules were close to the cell cortex 8
  2. Dynein does not reside on plus-ends of growing microtubules 8
  3. The number of dynein spots is larger than the number of microtubule plus ends 8
  4. Alternative mechanism for dynein following the plus ends of shrinking microtubules, which are not in contact with the cell cortex 9
  5. Occasional events during the oscillations: microtubule breakage and detachment 9
  6. Analysis of the SPB velocity 10
  7. The number of dyneins on the leading and the trailing microtubules 11
  8. Switch-like redistribution of dynein 11
- B. Cell preparation, growth and media 11
- C. Zygote (meiosis) induction 11
- D. Strains and gene tagging 12
  1. Triple GFP tagging of dhc1 12
  2. mCherry tagging of alpha2-tubulin (atb2) and subsequent integration into the ars1 locus of the strain SV56 13
- E. Time-lapse fluorescence microscopy 14
- F. Laser ablation 15
- G. Image analysis 15
  1. Spindle pole body tracking in the strain SV28xSV31 expressing Sid4-GFP and GFP-atb2 (Figs. 1 and 6) 15
  2. Color merge of images from the strain SV81 expressing Dhc1-3GFP and mCherry-tubulin (Figs. 2 and 3) 15
  3. Kymograph (Fig. 2E) 15
  4. Line profiles (Fig. 3 and S8C, D) 15
  5. Analysis of dynein dynamics and SPB tracking in the strain SV81 expressing Dhc1-3GFP and mCherry-tubulin (Fig. 5) 15
- H. Supplemental Movie Legends 15
  1. Supplemental Movie 1: Spindle pole body (SPB) movement via microtubules 15
  2. Supplemental Movie 2: Laser ablation of the leading microtubules affects the SPB movement 15
  3. Supplemental Movie 3: Laser ablation of the trailing microtubules does not affect the SPB movement 16
  4. Supplemental Movie 4: Laser ablation of the tip of the leading microtubules does not affect the SPB movement 16
  5. Supplemental Movie 5: Dynein signal is stronger on the leading than on the trailing microtubules 16
  6. Supplemental Movie 6: Dynein is distributed in a spotted pattern along the leading microtubules 16
  7. Supplemental Movie 7: Dynein remains on the end of depolymerizing microtubules 16

Additional references 17

---

*These authors contributed equally to this work

I. THEORY

A. Minimal model

In our one-dimensional model, the position of the SPB along the long axis of the cell is denoted x_{SPB} (Fig. 4). The two cell ends are located at $x = -L/2$ and $x = L/2$, respectively, where L is the cell length. Microtubules can grow at either side of the SPB; for simplicity, we first consider a single microtubule on each side. The length of the microtubule growing to the left is denoted L_ℓ, its plus-end is located at $x_\ell = x_{SPB} - L_\ell$. Similarly, the plus-end of the rightward growing microtubule of length L_r is located at $x_r = x_{SPB} + L_r$. Microtubules can be in one of two states: growing with velocity v_g or shrinking with velocity v_s. Catastrophe occurs when the plus-end of a microtubule reaches the cell end. Subsequently, the microtubule shrinks until its length vanishes; this is followed by nucleation of a growing microtubule.

The force balance of viscous friction force and the forces F_ℓ and F_r acting on the left and the right microtubule, is given by

$$\xi \frac{\mathrm{d}x_{SPB}}{\mathrm{d}t} = F_\ell + F_r \tag{S1}$$

where ξ is the friction coefficient of the system consisting of the nucleus, SPB, and microtubules. The forces F_ℓ and F_r are exerted by attached motors,

$$\begin{aligned} F_\ell &= N_\ell f_\ell \\ F_r &= N_r f_r . \end{aligned} \tag{S2}$$

Here, N_ℓ and N_r are the total number of motors attached to each microtubule and linked to the cortex, and f_ℓ and f_r the forces generated by a single motor on the left and the right microtubule, respectively. The forces f_ℓ and f_r are described by linear force-velocity relationships:

$$\begin{aligned} v &= v_0(1 + f_\ell/f_0) \\ v &= v_0(-1 + f_r/f_0). \end{aligned} \tag{S3}$$

Here, the velocity of motors with respect to the microtubule is denoted v, the velocity in the absence of force v_0, and f_0 is the stall force of the motor. Assuming that microtubules are rigid, the velocity of the motor with respect to the microtubule is equal to the negative SPB velocity

$$v = -v_{SPB} = -\frac{\mathrm{d}x_{SPB}}{\mathrm{d}t}. \tag{S4}$$

By combining Eqs. (S1), (S3), and (S4), the force balance reads:

$$\frac{\mathrm{d}x_{SPB}}{\mathrm{d}t} = v_0 \frac{f_0(N_r - N_\ell)}{f_0(N_r + N_\ell) + \xi v_0}. \tag{S5}$$

Eq. (S5) implies that there is no movement when the total number of motors on the left and the right microtubule is equal ($N_\ell = N_r$). On the other hand, if the number of motors on one side is significantly larger than on the other, e.g. $N_r \gg N_\ell$, and $N_r \gg \xi v_0/f_0$, the SPB velocity is close to v_0. Note that the SPB velocity cannot exceed v_0.

The linear densities, $n_\ell = N_\ell/L_\ell$ and $n_r = N_r/L_r$, of the motors attached to the left and the right microtubule, respectively, obey the kinetic equations

$$\begin{aligned} \frac{\mathrm{d}n_\ell}{\mathrm{d}t} &= k_{on}c - k_{off}(-f_\ell)n_\ell \\ \frac{\mathrm{d}n_r}{\mathrm{d}t} &= k_{on}c - k_{off}(f_r)n_r, \end{aligned} \tag{S6}$$

which describe attachment and detachment of motors. Here, c denotes cytoplasmic concentration of motors and k_{on} characterizes the rate of attachment of motors to microtubules. The motor detachment rate, k_{off},

$$k_{off}(f) = k_0 \exp(f/f_c) \tag{S7}$$

depends on the load force acting on individual motors. Here, k_0 is the detachment rate in the absence of load, and f_c a characteristic force. By combining Eqs. (S3), (S4) and (S7), the detachment rates can be written as

$$k_{off} = k_0 \exp\left[\frac{f_0}{f_c}\left(1 \pm \frac{v_{SPB}}{v_0}\right)\right], \tag{S8}$$

where the plus and the minus sign correspond to the left and right microtubule, respectively. Note that in Eq. (S8) the detachment rate k_{off} depends only on the velocity, v_{SPB}, that can be measured *in vivo*, unlike the load force in Eq. (S7).

B. The influence of parameter changes on the minimal model

The model can generate oscillations, as shown in Figs. S1A-C. In order to identify the region of the parameter space where oscillations arise, we varied three parameters for which there are no experimentally measured values, k_0, $k_{on}c$, and f_c. When the detachment rate in the absence of load, k_0, was decreased or increased 10 times, with respect to the value used in the main text ($k_0 = 0.01$ s^{-1}), the behavior of the model did not change considerably (compare Fig. S1A-f with Fig. 5A-C).

The attachment rate per unit length, $k_{on}c$, defines how fast the motors accumulate on the microtubule. We varied this parameter from 0 to 0.14 μm^{-1}s^{-1}, while keeping all the other parameters constant at values used in the main text (Fig. 4G). The model generates oscillations for all values of $k_{on}c$ except for $k_{on}c = 0$ (Fig. S2). With increasing $k_{on}c$, the curve with $f_c = 1$ pN (green) shows a sharp transition from oscillations with small amplitudes to those with large amplitudes at $k_{on}c \approx 0.073$ μm^{-1}s^{-1}, while the other three curves ($f_c = 2$ pN, black,

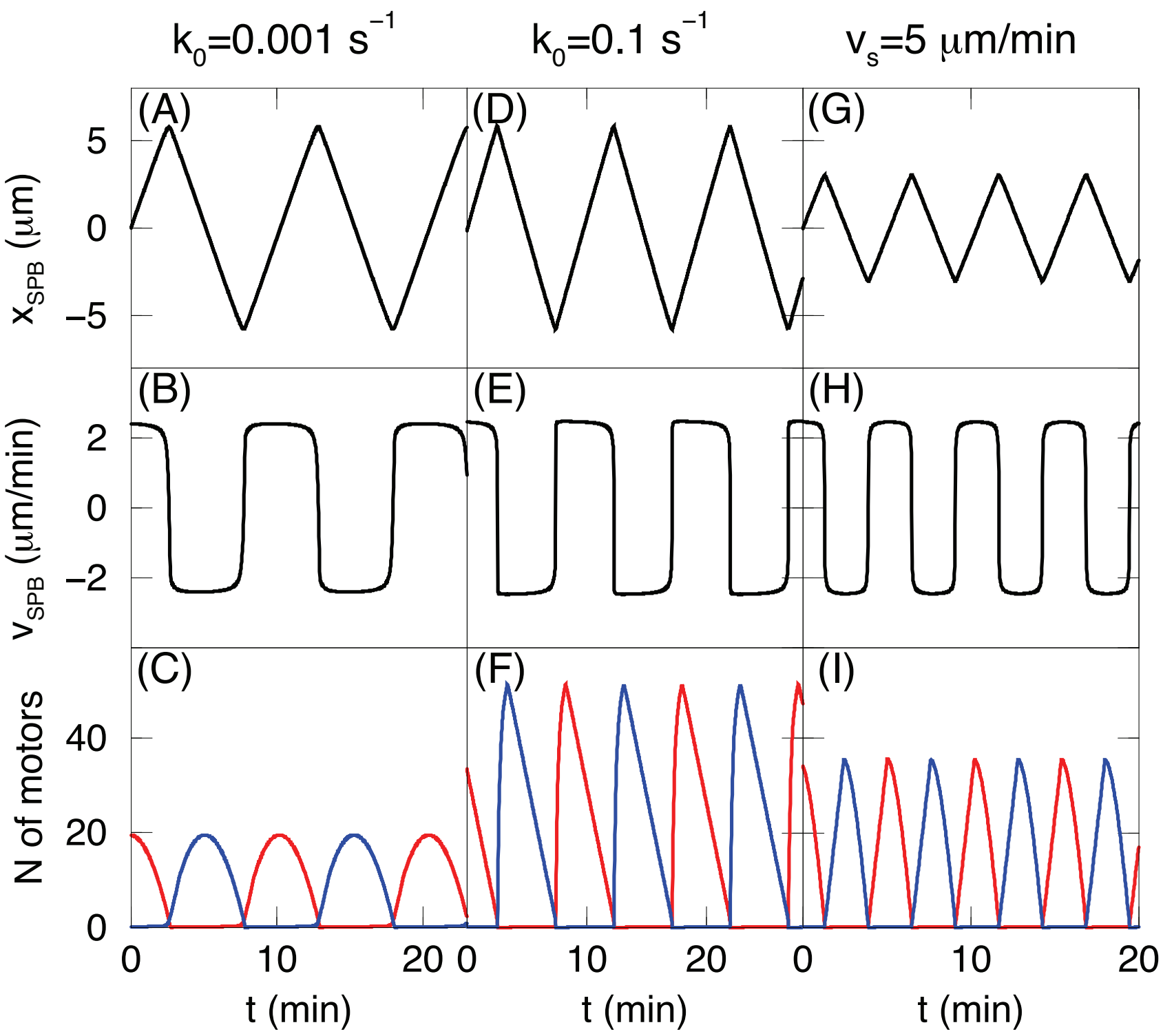

Figure S1: Model behavior for different values of parameters. (A, D, G) Position of the SPB; (B, E, H) velocity of the SPB; (C, F, I) the number of motors attached to the left microtubules (blue) and the right microtubules (red) as a function of time, for $k_0 = 0.001$ s^{-1}, $k_0 = 0.1$ s^{-1}, and $v_s = 5$ μm/min. In order to obtain roughly the same number of attached motors, we chose $k_{on}c = 0.02$ μm^{-1}s^{-1} in (A-C), $k_{on}c$=0.5 μm^{-1}s^{-1} in (D-F), and $k_{on}c$=0.1 μm^{-1}s^{-1} in (G-I). In each case, all the remaining parameters are as in Fig. 4G.

$f_c = 3$ pN, red, and $f_c = 100$ pN, blue) show a smooth increase in amplitude (Fig. S2). For $f_c = 1$ pN and $0 < k_{on}c \lessapprox 0.073$ μm^{-1}s^{-1} the amplitude is about 50 times smaller than the experimentally observed one. At $k_{on}c \approx 0.073$ μm^{-1}s^{-1}, there is a sharp transition to oscillations with amplitude values similar to those seen in experiments.

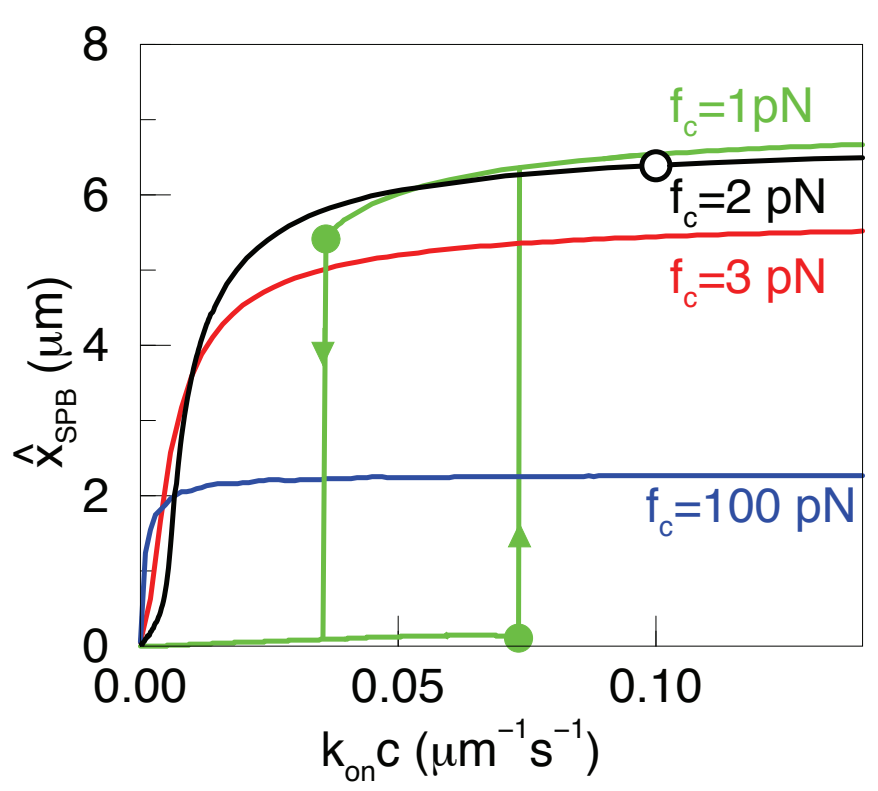

Figure S2: Bifurcation diagram for the minimal model. The amplitude, $\hat{x}_{SPB}$, of the oscillations is plotted as a function of $k_{on}c$ for four different values of f_c. The hollow circle marks the position in the parameter space corresponding to Figs. 4 and 5. For $f_c = 1$ pN, hysteresis occurs at $k_{on}c \approx 0.035-0.073$ μm^{-1}s^{-1}.

In general, there are two regimes which can be defined according to the value of the characteristic force f_c. For $f_c \lessapprox 4$ pN, the number of motors on the trailing microtubule is significantly smaller than the number of motors on the leading microtubule, due to the fast detachment caused by load (Fig. S3 A-C). Therefore, we call this regime *strong load-dependence* regime. For $f_c \gtrapprox 10$ pN, the number of motors on the trailing microtubule is comparable to the number of motors on the leading microtubule (Fig. S3 D-F). We call this regime *weak load-dependence* regime. By increasing f_c from 4 pN to 10 pN, a smooth transition between these two regimes occurs.

The stall force of dynein, $f_0 = 7$ pN, was taken from [S1], [S2]. However, another group measured $f_0 = 1.1$ pN [S3]. Using the latter value did not change the behavior of the model. Note that when f_0 is scaled, f_c and $k_{on}c$ have to be scaled accordingly in order to maintain the functional dependence in Eq. (S8) and the total force exerted by the motors attached to each microtubule in Eq. (S2) ($f_c = 0.34$ pN and $k_{on}c = 0.636$ μm^{-1}s^{-1}).

We next varied the three parameters v_g, v_s, and v_0, which we have measured, but they show variation between cells of the same strain, as well as between strains

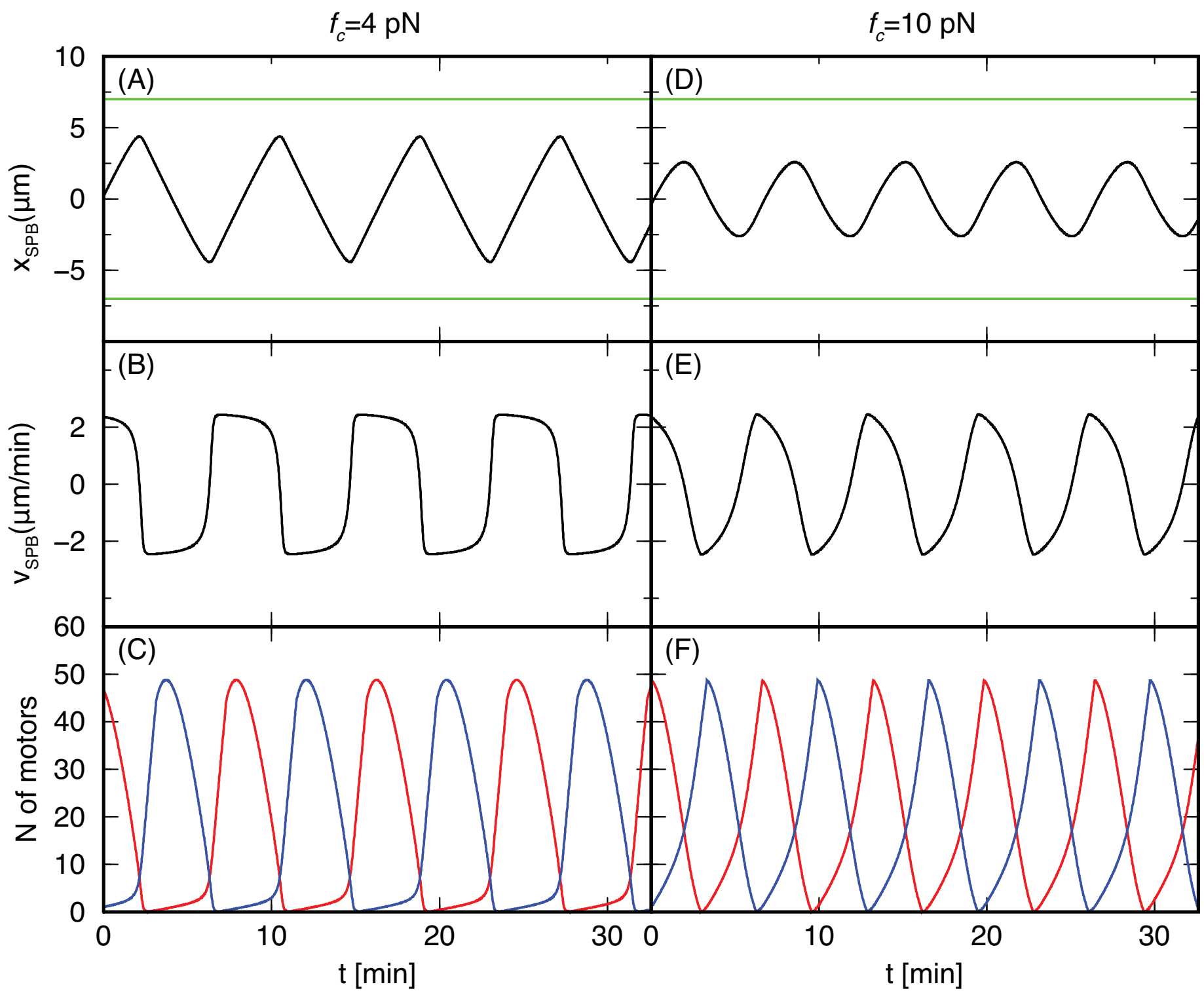

Figure S3: Behavior of the model in strong load-dependence regime (A-C) $f_c = 4$ pN, and weak load-dependence regime (D-F) $f_c = 10$ pN. (A, D) Position and (B, E) velocity of the SPB; (C, F) number of motors attached to the left microtubules (blue) and the right microtubules (red) as a function of time. In each case, all the remaining parameters are as in Fig. 4G.

Table SI: SPB velocities for 4 different strains, measured as described in Section II A 6 and illustrated in Fig. S7.

Strain	mean ± s.d. [μm/min]	N of half-periods	N of cells
KT233	5.65 ± 1.37	133	15
SV28xSV31	4.98 ± 2.23	20	8
JW785	4.11 ± 1.16	17	6
SV81	2.84 ± 0.74	69	22

(Tables SI, SII, and SIII). Changing these parameters by the same factor affected only the period of the oscillations. Thus, we varied v_g and v_s, while keeping v_0 constant. All the other parameters had the values shown in Fig. 4G.

A change in v_g from 2.0 μm/min to values between 0.5 and 3.0 μm/min did not change significantly the behavior of the model. Since values of $v_g \gg v_0$ were not observed experimentally, we did not explore that part of the parameter space.

Additionally, polymerization rate (v_g) of the trailing microtubule may influence the behavior of dynein at the plus end of this microtubule. In particular, when the growth of the trailing microtubule is slower than the minus end-directed dynein velocity ($v_g < v_0$), load on dynein at the plus end of the trailing microtubule may increase. However, our experiments showed that dynein signal on the plus end of the trailing microtubules was similar to the dynein signal along these microtubules (Fig. 2A). Therefore, our model does not describe dynein at the plus of the trailing microtubule separately.

An increase in v_s from 2.5 μm/min to 5 μm/min resulted in a decrease of the amplitude and the period of the oscillations (Fig. S1G), while the waveforms (Fig. S1G-I) did not change substantially. However, choosing v_s to be significantly different than v_0 is not biologically relevant, since these two values were always similar in individual cells (Tables SII and SIII). We speculate that the dyneins linking the plus-end of the microtubule to the cell cortex stabilize the position of this end with respect to the cortex by exerting force on this end of the microtubule.

We performed the same parameter changes in the weak load-dependence regime ($f_c = 100$ pN), keeping all the other parameters as in Fig. 4G. The resulting oscillations are characterized by a number of motors attached to the trailing microtubule comparable to the number of motors attached to the leading microtubule, and SPB position as function of time close to a sinusoidal waveform (data not shown).

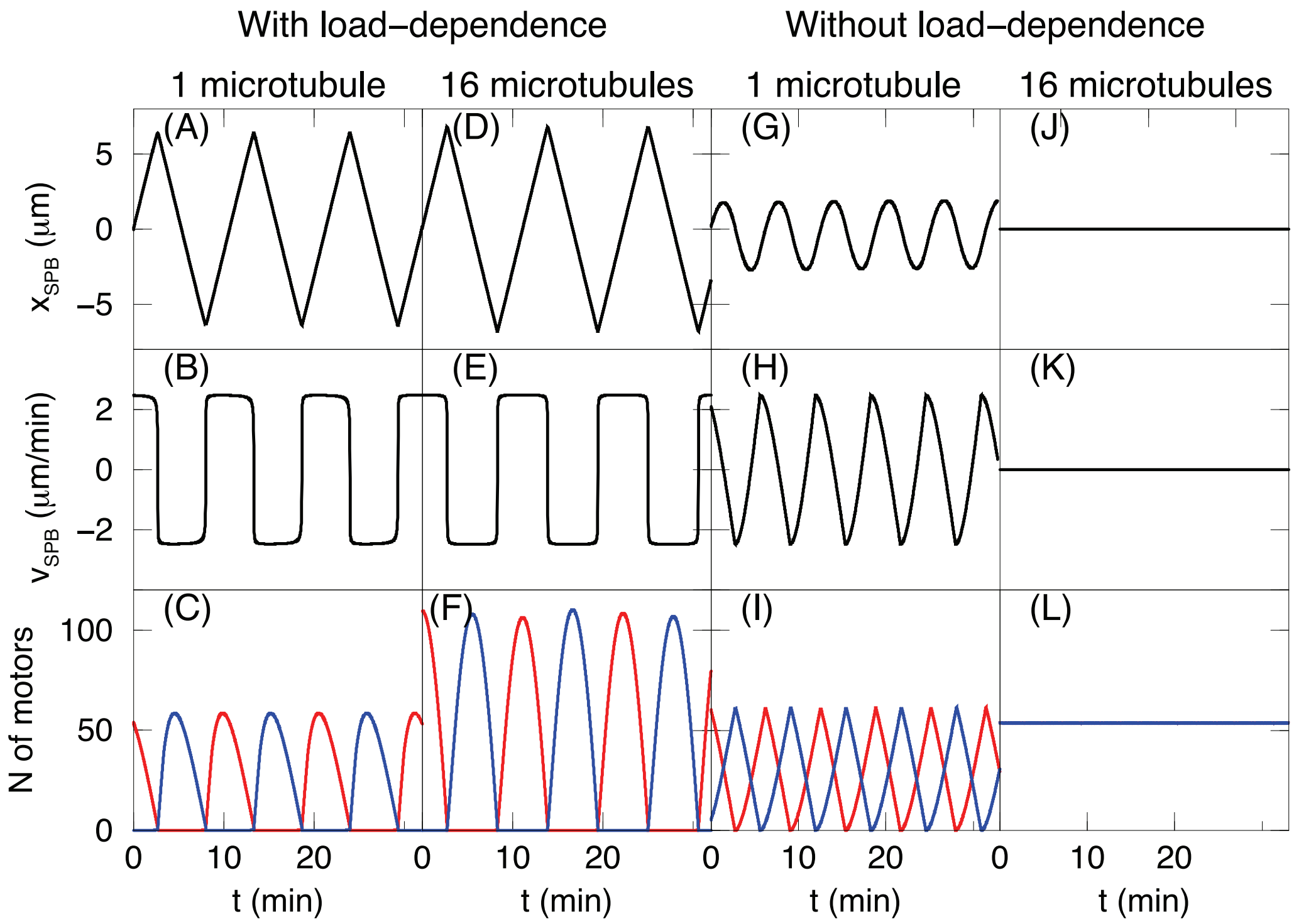

Figure S4: Influence of the load-dependence on the behavior of the model with a single and with multiple microtubules. (A, D, G, J) Position of the SPB; (B, E, H, K) velocity of the SPB; (C, F, I, L) the number of motors attached to the left microtubules (blue) and the right microtubules (red) as a function of time. The parameters for the model with single microtubules are as in Fig. 4G. For the model with multiple microtubules, T_{nucl} = 40 s, $k_{on}c$ =0.01 μm^{-1}s^{-1}, while the remaining parameters are as in Fig. 4G. In the model with multiple microtubules, the average number of microtubules on each side is 16.

C. Analytical solution of the minimal model

In order to understand the typical behavior of the model, we consider the simple limit where the SPB moves with the maximal motor velocity, $dx_{SPB}/dt = \pm v_0$. In this limit, the oscillations have an exact triangular waveform of amplitude of $L/2$.

For the leading microtubule, $k_{off}L/v_0 \ll 1$, thus the linear density of motors is described by $dn/dt \approx k_{on}c$. The total number of motors on this microtubule, N, is a product of the linear density of motors, n, and the microtubule length, $L_{MT} = L_0 - v_s t$, both of which depend linearly on time (the constant L_0 depends on the initial conditions). Consequently, the time-course of the total number of motors, $N = k_{on}c(L_0 - v_s t)t$, is a parabola (Figs. S1, S4). For the trailing microtubule, $k_{off}L/v_0 \gg 1$, thus the linear density of motors has a constant value ($dn/dt \approx 0$), which is much smaller than the average density of motors on the leading microtubules.

D. Multiple microtubules

For simplicity, the minimal model describes two microtubules, one on each side. The generalization to several microtubules is straightforward; it introduces a constant time interval for microtubule nucleation T_{nucl}. Unlike in the minimal model, a complete depolymerization of a microtubule is not followed by nucleation of a new microtubule.

The total numbers of microtubules on each side, M_ℓ and M_r for the left and the right side, respectively, are dynamically regulated. The total numbers of motors on each side are $N_\ell = \sum_{i=1}^{M_\ell} N_{\ell,i}$ and $N_r = \sum_{j=1}^{M_r} N_{r,j}$, where $N_{\ell,i}$ and $N_{r,j}$ are the total number of motors attached to the i-th microtubule on the left and to the j-th microtubule on the right side, respectively. The Eqs. (S6) are replaced by

$$\begin{aligned} \frac{\mathrm{d}n_{\ell,i}}{\mathrm{d}t} &= k_{on}c - k_{off}(-f_\ell)n_{\ell,i} \\ \frac{\mathrm{d}n_{r,j}}{\mathrm{d}t} &= k_{on}c - k_{off}(f_r)n_{r,j}, \end{aligned} \tag{S9}$$

where at the moment of microtubule nucleation, $n_{\ell,i} = n_{r,j} = 0$. Here, $n_{\ell,i} = N_{\ell,i}/L_{\ell,i}$ and $n_{r,j} = N_{r,j}/L_{r,j}$ are the linear densities of the motors on the i-th and the j-th microtubule on the left and the right side, respectively. The behavior of the model with multiple microtubules is shown in Fig. S4.

Table SII: Microtubule shrinkage rates, v_s, growth rates, v_g, and SPB velocities, v_0 (measured in the same time intervals as v_s), in the strain SV81.

Strain SV81		mean [μm/min]	s.d. [μm/min]	N
Shrinkage rate of leading microtubules attached to the cortex	Cell2	2.18	0.54	4
	Cell4	3.09	0.35	4
	Cell5	2.19	0.41	4
	Pooled (total)	2.49	0.60	12
SPB velocity	Cell2	2.36	0.06	4
	Cell4	3.21	0.20	4
	Cell5	2.10	0.53	4
	Pooled (total)	2.56	0.58	12
Growth rate of trailing microtubules	Cell2	2.37	0.31	4
	Cell4	1.80	0.69	4
	Cell5	1.82	0.85	4
	Pooled (total)	1.99	0.65	12
Shrinkage rate of leading microtubules not attached to the cortex	Cell2	3.04	0.87	4
	Cell4	3.16	0.99	4
	Cell5	4.19	1.65	4
	Pooled (total)	3.46	1.23	12

Table SIII: Microtubule shrinkage rates, v_s, growth rates, v_g, and SPB velocities, v_0 (measured in the same time intervals as v_s), in the SV28xSV31 strain crossing.

Strain SV28xSV31		mean [μm/min]	s.d. [μm/min]	N
Shrinkage rate of leading microtubules attached to the cortex	Cell1	5.95	1.32	4
	Cell2	4.32	1.27	3
	Cell6	2.77	0.64	2
	Pooled (total)	4.70	1.70	9
SPB velocity	Cell1	6.13	0.85	4
	Cell2	4.62	0.83	3
	Cell6	4.21	1.97	2
	Pooled (total)	5.20	1.32	9
Growth rate of trailing microtubules	Cell1	3.73	0.77	5
	Cell2	2.66	0.77	3
	Cell6	3.16	0.74	2
	Pooled (total)	3.30	0.83	10

E. Sensitivity of the model to load-dependence

We investigated whether load-dependence of detachment is required to generate oscillations. Both models with a single and multiple microtubules generate oscillations of the same type (compare Fig. S4A-C with Fig. S4D-F). When the load-dependence of motor detachment is excluded from the model with a single microtubule, $k_{off} = k_0$, oscillations still occur, but the waveforms are qualitatively different from the ones obtained with load-dependence (Fig. S4G-I). An even larger change in the model behavior results from excluding the load-dependence from the model with multiple microtubules; no oscillations exist in this case (Fig. S4J-L).

F. Two-step attachment and detachment of dynein

In our minimal model, the motors simultaneously attach to and detach from microtubules and the cell cortex. This is, however, most likely a complex process involving two major steps (Fig. 4F in the main text). Our observation of dynein dynamics revealed that when the links between the microtubules and the cortex break, dynein detaches from the cortex and remains on the microtubules. This suggests that the dynein attached both to the microtubule and to the cortex detaches in two steps: in the first step, it detaches from the cortex and remains on the microtubule, while in the second step it detaches from the microtubule and is released into the cytoplasm. We assume that the attachment occurs in the same two steps, but in the opposite order.

We generalized our model to include a two-step attachment and detachment process. The motors on a microtubule are divided into two populations: those attached only to the microtubule (denoted by the additional index MT), and those attached both to the microtubule and the cortex (denoted by MTC). The total linear density of motors on the left microtubule is $n_\ell = n_{\ell,MT} + n_{\ell,MTC}$, where $n_{\ell,MT}$ is the linear density of the motors attached only to the microtubule, and $n_{\ell,MTC}$ the linear density of the motors attached both to the microtubule and the cortex. The following equations describe the two-step attachment and detachment kinetics:

$$\begin{aligned}
\frac{\mathrm{d}n_{\ell,MT}}{\mathrm{d}t} &= k_{on}^{cyt\to MT} c + k_{off}^{MTC\to MT}(-f_\ell) n_{\ell,MTC} \\
&\quad - k_{off}^{MT\to cyt} n_{\ell,MT} - k_{on}^{MT\to MTC} n_{\ell,MT} \\
\frac{\mathrm{d}n_{\ell,MTC}}{\mathrm{d}t} &= k_{on}^{MT\to MTC} n_{\ell,MT} \\
&\quad - k_{off}^{MTC\to MT}(-f_\ell) n_{\ell,MTC}. \qquad \text{(S10)}
\end{aligned}$$

Here, $k_{on}^{cyt\to MT} c$ describes the attachment rate to the microtubule, $k_{off}^{MTC\to MT}(-f_\ell)$ is the detachment rate from the cortex, which is load-dependent, $k_{off}^{MT\to cyt}$ is the detachment rate from the microtubule, and $k_{on}^{MT\to MTC}$ the attachment rate to the cortex. The load-dependence of the detachment rate from the cortex, $k_{off}^{MTC\to MT}(-f_\ell)$, is described by Eq. (S7). The parameters $k_{on}c$ from Eq. (S6) and k_0 and f_c from Eq. (S7) have a similar meaning here as in the minimal model. The two-step model therefore has two additional parameters, $k_{on}^{MT\to MTC}$ and $k_{off}^{MT\to cyt}$. Eq. (S10) will describe the dynein kinetics on the right microtubule, if we replace ℓ with r and $-f_\ell$ with f_r. Since in our experiments we did not observe dynein attached to the cortex, without being simultaneously attached to the microtubule, we do not consider this possibility in the model.

The values of $k_{on}c$, k_0, and f_c are the same as in the

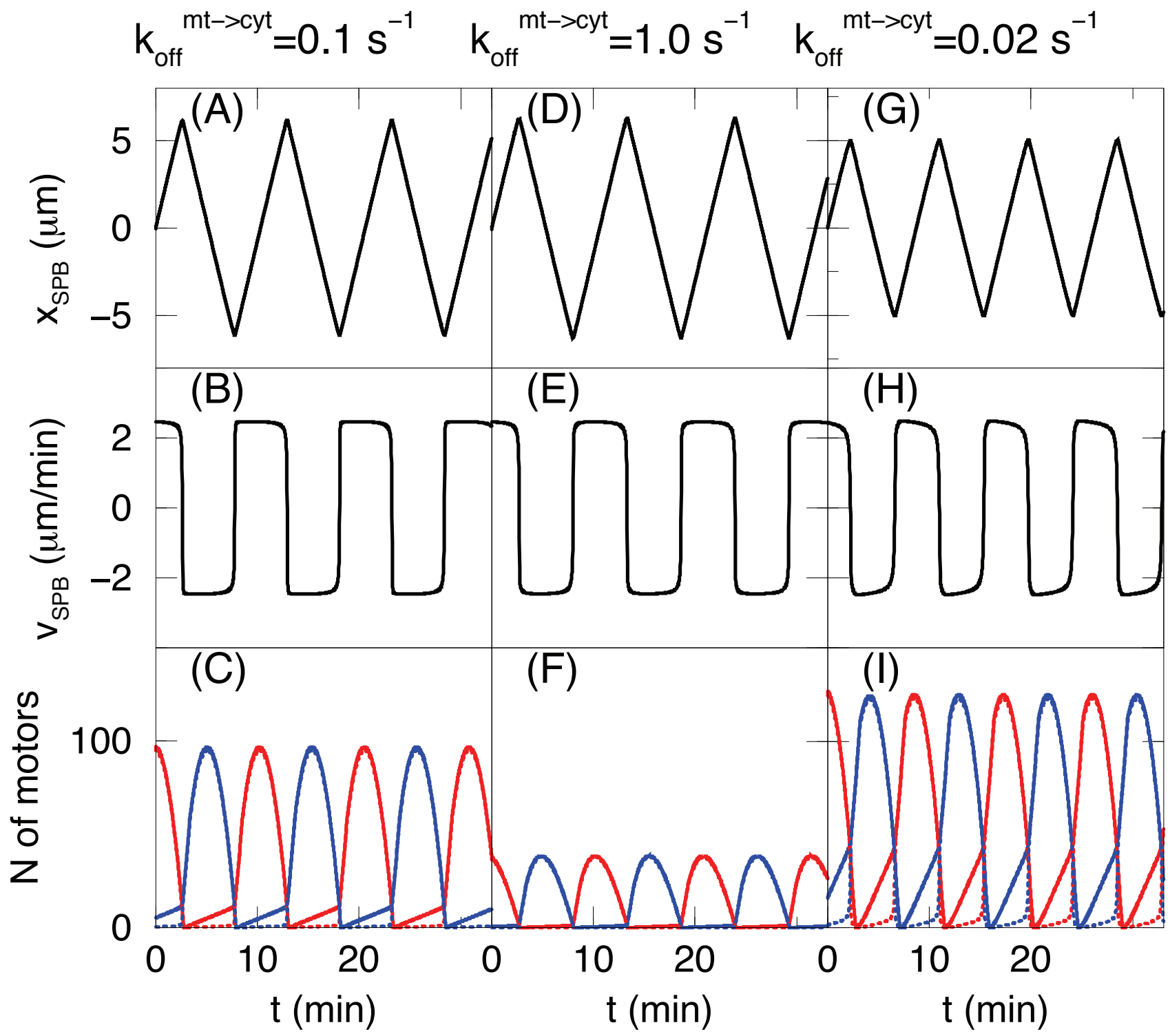

Figure S5: Two-step attachment and detachment of dynein. (A, D, G) Position of the SPB; (B, E, H) velocity of the SPB; (C, F, I) the total number of motors attached to the left microtubules ($n_\ell \cdot L_\ell$, full blue line) and the number of motors attached both to the cortex and to the left microtubules ($n_{\ell,MTC} \cdot L_\ell$, dotted blue line); the total number of motors attached to the right microtubules ($n_r \cdot L_r$, full red line) and the number of motors attached both to the cortex and to the right microtubules ($n_{r,MTC} \cdot L_r$, dotted red line). The results are shown for $k_{on}^{MT\to MTC} = 1.0$ s^{-1} and for three values of $k_{off}^{MT\to cyt}$. All the remaining parameters are as in Fig. 4G.

minimal model. We chose $k_{off}^{MT\to cyt} = 0.1$ s^{-1}, similar to the value of dynein run length (∼20 s) measured by Reck-Peterson *et al.* [S4]. The value of $k_{on}^{MT\to MTC}$ was 1 s^{-1} because the process of attachment of microtubule-bound dynein to the cortex is most likely faster than the release of microtubule-bound dynein into the cytoplasm, favoring accumulation of dynein at the microtubule-cortex interaction sites.

The behavior of the two-step model (Fig. S5) with the above parameters was similar to that of the minimal model. Increasing or decreasing $k_{on}^{MT\to MTC}$ while keeping $k_{off}^{MT\to cyt}$ constant did not have a substantial effect (not shown). Similarly, increasing $k_{off}^{MT\to cyt}$ while keeping $k_{on}^{MT\to MTC}$ constant did not affect the model behavior (Fig. S5D-F). As expected, decreasing $k_{off}^{MT\to cyt}$ resulted in an increase of the total number of motors on the trailing microtubule (Fig. S5I). Our experiments showed a small number of dynein on the trailing microtubule (Fig. 5F), consistent with Fig. S5C and F, i.e., $k_{off}^{MT\to cyt} = 0.1$-1 s^{-1}.

Since the generalization by introducing two-step dynein attachment and detachment did not change the behavior of the model significantly, we conclude that the one-step description, used in the minimal model (Section I A and main text), is a good approximation of this complex attachment/detachment process.

G. Nonlinear force-velocity relationship

Our minimal model includes a linear force-velocity relationship (Eq. (S3)), as a simple approximation of the measured curve [S1]. To test the effect of this approximation, we generalized our model by introducing a nonlinear force-velocity relationship, described by

$$f_r = 7.92 - 3.27 \cdot v_{SPB} - 2.4 \cdot \exp\left[-\frac{(v_{SPB} - 0.57)^2}{0.385}\right], \qquad \text{(S11)}$$

where v_{SPB} is expressed in μm/min and f_r in pN. We obtained this function by fitting the data from Fig. 1E in Ref. [S1]; we chose the fitting function to be the sum of a gaussian and a linear function. The resulting fit was scaled to the maximal velocity measured in our experiments, giving the final force-velocity relationship in Eq. (S11). For the typical values of the parameters (Fig. 4G), the behavior of the model is not affected by introducing this nonlinearity. This result is not surpris-

ing, since v_{SPB} is most of the time close to its maximum value, where nonlinearities in the force-velocity relationship have only a minor influence.

H. Differences between our model and other models involving microtubule pulling forces from the cortex

Our model differs from the models for the spindle oscillations in the one-cell stage *C. elegans* embryo in: 1) microtubule length-dependent attachment of dynein, 2) dynamic redistributions of dynein via the cytoplasm (see Discussion in the main text), and 3) a novel restoring force.
For oscillations to occur, a restoring force is needed which counteracts the outward movement. Previous studies proposed additional restoring forces for spindle oscillations based on microtubule pushing and aster deformation [S5], [S6]). Our model does not rely on additional restoring forces that would move the SPB towards the cell center. Instead, we provide a novel restoring mechanism, which is intrinsic to our model and based on microtubule length-dependent attachment of dynein.

II. EXPERIMENTS

A. Supplementary experimental results

1. The asymmetry of the dynein distribution was observed also when the trailing microtubules were close to the cell cortex

One can argue that the asymmetry of the dynein distribution between the leading and the trailing microtubules is established by the typical position of microtubules within the cell, where only parts of the trailing microtubules are close to the cell cortex and consequently to the attachment sites for dynein. However, the time-lapse sequence of Figure S6 demonstrates that the asymmetry in the dynein distribution did persist even when the trailing microtubules were in contact with the cell cortex (arrowheads, bent line and arrow). The bent trailing microtubule bundle (bent line), following the curved shape of the zygote, indicated that the trailing microtubules were in close contact with the cell cortex (arrowheads and bent line, see also Supplemental Movies 5 and 6). Furthermore, contact of the tip of the trailing microtubules with the cell end (arrow) did not influence the dynein pattern. Taken together, these findings indicate that the asymmetry of the dynein distribution indeed results from load-dependent detachment rather than from the microtubule position relative to the cortex.

2. Dynein does not reside on plus-ends of growing microtubules

The signal on the leading microtubules was distributed in a spotted pattern (Figure 2B; Supplemental Movie 6). Dynein spots have been observed in budding yeast mitosis, where they move away from the SPB on the growing plus-ends of astral microtubules and become off-loaded at the cortex [S7]. To the contrary, movement of dynein spots away from the SPB was not observed in our experiments (Figure 2). One can argue that dynein spots were not observed because dynein may have been distributed on a large number of growing plus-ends. This is, however, unlikely since we observed 1-2 growing microtubules on the leading side. The number of microtubules was estimated by using the plus-end tracking protein Mal3p labeled with GFP [S8] (n = 6 periods in 4 cells; Figure 3E). We conclude that in meiotic prophase in fission yeast, dynein does not reside on plus-ends of growing microtubules.

3. The number of dynein spots is larger than the number of microtubule plus ends

We asked whether the observed dynein spots (Figure 2) represent dynein clusters only at the plus ends of individual microtubules or also laterally along the microtubules. We observed a larger number of dynein spots than of microtubule plus ends, suggesting that dynein resides along the lateral sides of microtubules in addition to the plus-ends. The number of dynein spots was measured directly from the images (Figure 3A, 3C; Figure S8), while the number of microtubule plus-ends was estimated as described below.
In the example shown in Figure 3A, the constant tubulin signal along the leading microtubules represents most likely two equally long microtubules (Figure 3C), based on the following arguments. Unlike in meiotic prophase, the number of microtubules in interphase is known: within a bundle, typically a single microtubule reaches the cell tip [S9] [S10]. To determine the number of microtubules in meiosis, we compared the relative signal of mCherry-labeled microtubule bundles with respect to the cytoplasmic mCherry-tubulin signal in cells in meiotic prophase with those in interphase, in the same field of view (Figure 3A and 3B; Figure S8). In the interphase cell, the signal of a single microtubule, which is found in the part of the bundle near the cell tip, was $\sim 50\%$ above the cytoplasmic signal (Figure 3D). The signal along the leading microtubules in the meiotic cell was $\sim 100\%$ above the cytoplasmic signal in this cell (Figure 3C). Assuming an equal concentration of non-labeled tubulin in these two cells, comparison between the relative signal of mCherry-labeled microtubules in these two cells suggests that there are two leading microtubules in the meiotic cell. This is an upper bound for the number of leading microtubules because the expression of non-

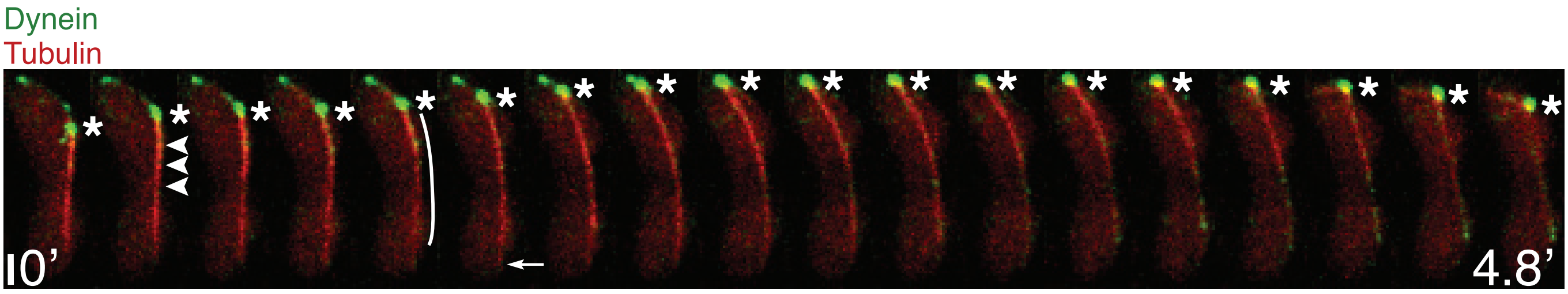

Figure S6: A time-lapse sequence of a cell expressing Dhc1p-3GFP (dynein; green) and mCherry-alpha2-tubulin (red) during the oscillations. The asymmetric dynein distribution did not change when the trailing microtubules were in close contact with the cell cortex. The arrowheads and the bent line indicate the close contact between the trailing MTs and the cell cortex. The arrow points to trailing MTs interacting with the cell end. The position of the SPB is marked with asterisks. The scale bar represents 2 μm.

labeled tubulin in meiotic prophase is lower than that in interphase [S11].
Independently, we determined the total number of microtubules in meiotic cells as a ratio between the nucleation and the catastrophe rate. By marking growing plus-ends using Mal3-GFP, we determined the nucleation rate as 3.5 ± 1.2 min^{-1} (mean $\pm$ s.d., n = 105 events in 3 cells; Figure 3E). Using the known catastrophe rate of 0.36 min^{-1} [S12], we estimated the total number of microtubules to be approximately ten. In the examples shown in Figure 3C and Figure S8D, the relative microtubule signal on the trailing side was roughly six times higher than that on the leading side. Thus we estimated that these cells had twelve microtubules on the trailing side and two on the leading side, which is consistent with the number of leading microtubules determined by comparison with interphase cells. In the example in Figure 3C, the strongest dynein signal along the leading microtubules was found at the point that corresponds to the plus-ends of both leading microtubules. There were, however, at least two additional dynein spots along these microtubules. We conclude that dynein is present along the microtubules and not only at their ends.

4. Alternative mechanism for dynein following the plus ends of shrinking microtubules, which are not in contact with the cell cortex

We observed occasional breakage of the dynein-mediated links between the plus end of the microtubules and the cortex. In these cases dynein, which was previously stationary with respect to the cortex, detached from the cortex and followed the plus end of the depolymerizing microtubules (Figures 2C and 2E; Figure S9; Supplemental Movie 7). In other fungal systems, dynein accumulation at the microtubule plus end requires a plus end-directed motor [S13] [S14]. Similarly here, the presence of dynein at the plus ends of shrinking microtubules which are not in contact with the cell cortex may be a consequence of transport by plus end-directed motors. Thus, after the plus end detaches from the cortex, dynein

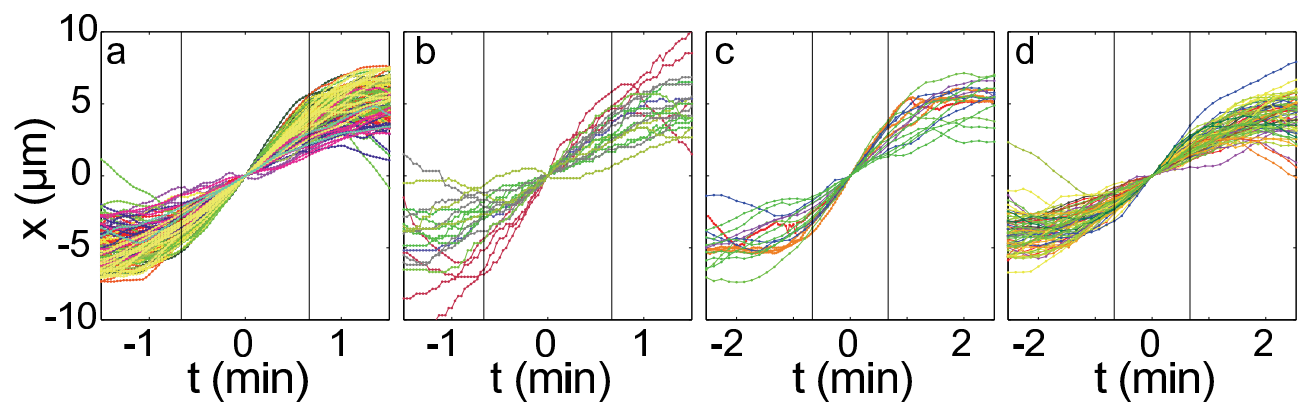

Figure S7: SPB position as a function of time for the strain (A) KT233 (Sid4-GFP), (B) SV28xSV31 (Sid4-GFP, GFP-Atb2), (C) JW785 (GFP-Dhc1), and (D) SV81 (Dhc1-3GFP, mCherry-Atb2). The vertical lines mark the fitting range. Each color represents a single cell. For details see Section II A 6.

observed at this end may be new dynein transported along the microtubules instead of the dynein that was linking the plus end to the cortex. To account for the observed constant dynein signal on the plus end before and after the plus end detachment from the cortex (within 10%; see Figure S9), transport by plus end-directed motors would imply detectable movement of dynein spots towards the plus end. Such movement was, however, not observed in our experiments (Figure 2 and Figure S9). We conclude that when the plus end detaches from the cortex, dynein also detaches from the cortex and remains on the microtubule.

5. Occasional events during the oscillations: microtubule breakage and detachment

Occasionally, we observed breakage of the leading microtubules (Figure S9A). In this example, the breakage occured near to the SPB (first white arrow), and subsequently the SPB reversed the direction of motion. After the breakage, the microtubule fragment depolymerized and dynein stayed attached to the depolymerizing microtubule fragment (white arrows).

In addition, we occasionally observed detachment of the leading microtubules from the cell cortex and their subsequent depolymerization (Figure S9B, see also

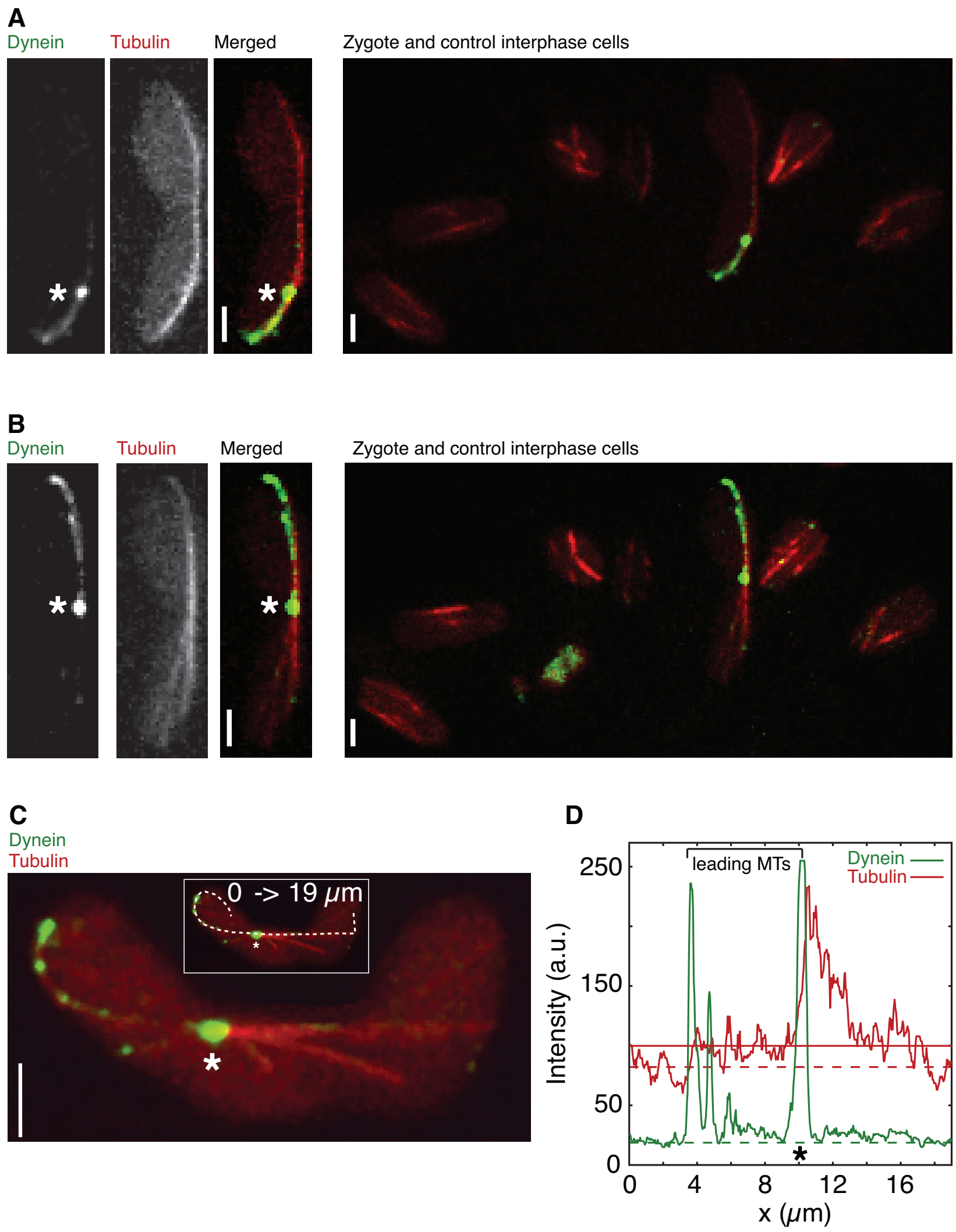

Figure S8: (A) and (B) show a zygote expressing Dhc1p-3GFP (dynein; green) and mCherry-alpha2-tubulin (red) at two different time points during the oscillations, together with interphase cells in the same field of view. Note the high amount of dynein distributed along the leading microtubules. (C) shows another zygote, also expressing Dhc1p-3GFP (dynein; green) and mCherry-alpha2-tubulin (red). (D) shows the intensity line profiles of dynein (green) and tubulin (red) measured along the white dashed line in (C). The estimated cytoplasmic intensity of dynein and tubulin are indicated by a dashed green and red line, respectively. The tubulin signal along the leading microtubules is constant (noisy solid red line shows the data, while the horizontal solid red line is the estimated signal corresponding to the leading microtubules) whereas the dynein signal (solid green line) shows several peaks along the same path (D), demonstrating that dynein is present along the microtubules and not only at their plus ends. Asterisks mark the SPB, vertical scale bars represent 2 μm.

Fig. 2C and E in the main text). After the detachment, dynein moved towards the SPB on the depolymerizing plus end of the microtubule (arrowheads). The SPB continued to move in the same direction, led by another leading microtubule. Altough the model does not account for them, these occasional events do not contradict the model.

6. *Analysis of the SPB velocity*

SPB velocity of four different strains (Table SI) was measured automatically from the SPB tracks, using a program written in Matlab. Time points when the SPB passes the cell center were identified (denoted by $t = 0$), and the velocities were found by a linear fit to the data of the SPB position with respect to time in the interval $t = \pm 2/3$ min, using only those parts of the SPB trajectory where the SPB covered a distance greater than 7 μm within ($t = \pm 1.5$) min (strains KT233 and SV28xSV31) or ($t = \pm 2.5$) min (strains JW785 and SV81), see Fig. S7.

Table SIV: Strains used in this study

Name	Genotype	Source	Microscope setup	Temperature
SV16	h^+ *sad1-CFP-kan*^{r} *kan*^{r}*-nmtP3-GFP-atb2*$^+$*leu1-32*	this study	2	25 ± 0.5 °C
SV17	h^- *sad1-CFP-kan*^{r} *kan*^{r}*-nmtP3-GFP-atb2*$^+$*leu1-32*	this study	2	25 ± 0.5 °C
SV28	h^+ *sid4-GFP-kan*^{r} *kan*^{r}*-nmtP3-GFP-atb2*$^+$ *ade6-M210 leu1-32 ura4-D18*	this study	1, 2, 3	25 ± 0.5 °C
SV31	h^- *sid4-GFP-kan*^{r} *kan*^{r}*-nmtP3- GFP-atb2*$^+$ *ade6-M210 leu1-32 ura4-D18*	this study	1, 2, 3	25 ± 0.5 °C
SV81	h^{90} *dhc1-3xGFP-kan*^{r} *ars1::LEU2-nmt1-mCherry-atb2*$^+$ *leu1-32 lys1 ura4-D18*	this study	1, 4	25 ± 0.5 °C
KT233	h^{90} *sid4-GFP-kan*^{r} *leu1-32*	K. Gould	1	25 ± 0.5 °C
SV56	h^{90} *dhc1-3xGFP-kan*^{r} *leu1-32 lys1 ura4-D18*	this study	1, 2	25 ± 0.5 °C
JW785	h^{90} *kan*^{r}*-nmtP3-GFP-dhc1 ade6-M216 leu1-32*	(YGRC Japan) M. Yamamoto	1	25 ± 0.5 °C
(FY15622) CRL152	h^{90} *leu1-32 lys1 ura4-D18*	(YGRC Japan) Y. Hiraoka	-	-
P3-GFP-atb2	h^- *kan*^{r}*-nmtP3-GFP-atb2*$^+$ *leu1-32*	T. Toda	-	-
KGY2679	h^+ *sid4-GFP-kan*^{r} *ade6-M210 leu1-32 ura4-D18*	K. Gould	-	-
JW820	h^+ *sad1-CFP-kan*^{r} *mid1-YFP-kan*^{r} *ade6-M210 leu1-32 ura4-D18*	T.D. Pollard	-	-

The velocities measured manually (Tables SII and SIII) were not significantly different than those measured automatically (Table SI, strains SV81 and SV28xSV31; *t*-test yielded $p = 0.2$ and 0.8, respectively).

The four strains from Table SI showed different SPB velocities, with the highest velocity found in the strain expressing Sid4-GFP, and the lowest in that expressing Dhc1-3GFP and mCherry-Atb2. In spite of the variability of the velocities, oscillations occurred in all the strains, emphasizing the robustness of this process.

7. *The number of dyneins on the leading and the trailing microtubules*

To extend the data analysis for Fig. 5F in the main text, we calculated the average curves for the SPB position and the number of dyneins on the leading and the trailing microtubules. As the SPB moved, the number of dyneins on the leading microtubules increased and then decreased, while the number of dyneins on the trailing microtubules remained small (Fig. S10).

8. *Switch-like redistribution of dynein*

We plotted the velocity of the SPB, v_{SPB}, as a function of the imbalance of the number of motors, ΔN, on each side of the SPB (Fig. S11). Both the experimental measurements (Fig. S11A) and the numerical calculation (Fig. S11B) yielded a sigmoidal curve. This directly shows the switch-like redistribution of dyneins from one side to the other via the cytoplasm *in vivo*, which is reproduced by the model.

B. Cell preparation, growth and media

The cells were grown either on Yeast Extract (YE) medium agar plates or on Edinburgh Minimal Medium (EMM) with appropriate supplements at 30°C. For imaging, the *nmt1*-promotor (strains SV28 and SV31) and therefore the GFP-alpha-tubulin2 (atb2) expression was partially repressed by adding 2 μM thiamine to the agar plates. All *S. pombe* strains used in this study are listed in table SIV.

C. Zygote (meiosis) induction

To induce sexual differentiation the cells were resuspended in a small amount of 0.85% NaCl solution (80-100 μl) in order to get a dense cell suspension and then spotted on a malt extract media agar plate (MEA). To induce meiosis in the heterothallic strains SV28 (mating type h$^+$) and SV31(mating type h$^-$) the cell types were mixed with an 1:1 ratio before spotting them on a MEA plate. Before imaging, the cells were grown overnight (12-14 hours) at 25°C on MEA plates to obtain zygotes that are in meiotic prophase. For microscopy, the cells were resuspended in liquid EMM lacking nitrogen (EMM-N) and transferred to a glass-bottom microwell dish (MatTek, Ashland) coated with 4 μl of 2 mg/ml lectin BS-1 (Sigma-Aldrich) in PBS for approximately 15 minutes to let them attach to the glass bottom. Non-immobilized cells were then washed out three times with EMM-N.

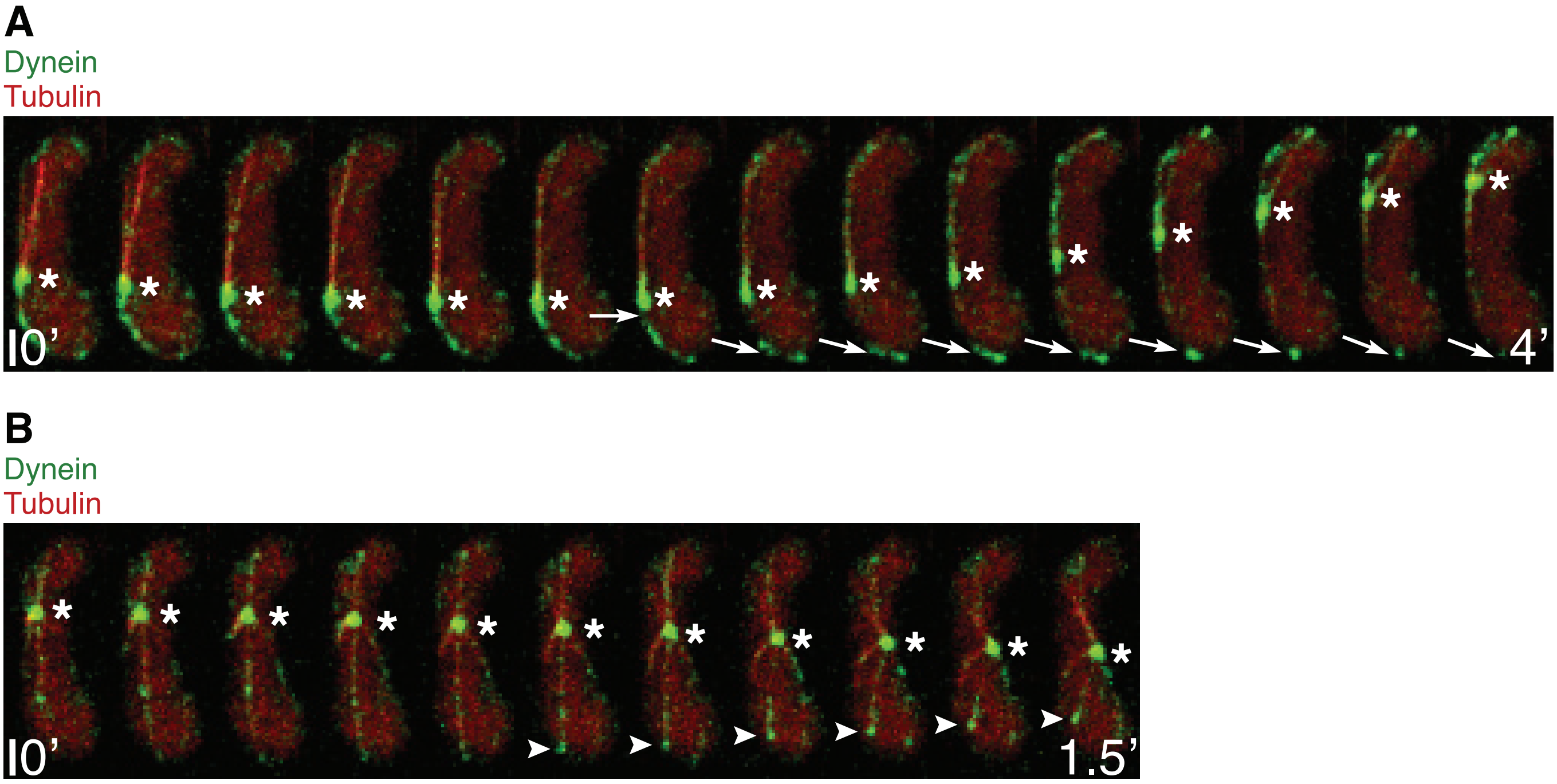

Figure S9: The image sequences are taken from Supplemental Movie 6 (A: frames 5-32 with a time interval of 16 s between the images; B: frames 121-131 with a time interval of 8 s between the images). The zygote expresses Dhc1p-3GFP (dynein; green) and mCherry-alpha2-tubulin (red). (A) shows a breakage of the leading microtubules. The first arrow marks the breakage of the microtubule, while the following arrows point to the depolymerizing fragment. Note that dynein stays attached to the depolymerizing microtubule fragment. (B) shows detachment of a leading microtubule from the cortex. The arrowheads point to the depolymerizing plus end of the microtubule. Note that dynein stays attached to the microtubule plus end. Asterisks indicate the position of the SPB. Scale bars represent 2 μm.

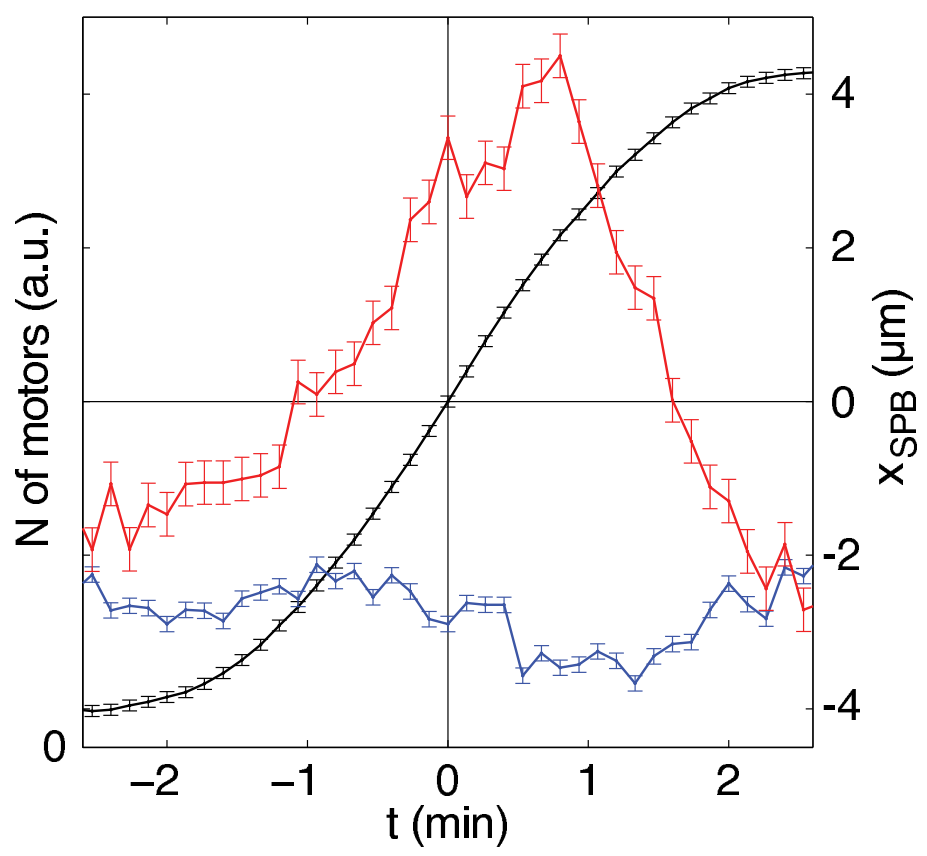

Figure S10: Experimental measurements of the SPB position (black), the number of motors on the leading microtubule (red), and on the trailing microtubule (blue). The number of motors is in arbitrary units, and was calculated from the dynein intensity. The data shows the mean value of 50 half-periods from 11 cells. The error bars represent s.e.m.

D. Strains and gene tagging

1. *Triple GFP tagging of dhc1*

Carboxy-terminal epitope tagging of Dhc1 with triple GFP was performed by using a polymerase chain reaction (PCR) gene-targeting method [S15] where the open reading frame (ORF) of the triple GFP construct was integrated into the original gene locus by homologous recombination. For these purposes the triple GFP sequence was PCR amplified from the pSL-3xGFP plasmid (kind gift from G. Steinberg) with flanking restriction sites PacI and AscI. The following primer pair was used:

1. Forward-Primer (SV-PacI-C-3xGFP-Forw)

5'-CGC TTA ATT AA C TTA CGA ATT CGA ATG G CC ATG -3'

(PacI: TTA ATT AA; pSL-3xGFP- sequence: TTA CGA ATT CGA ATG G; Start 3xGFP: CC ATG)

2. Reverse-Primer (Real-end-SV-AscI-3xGFP-Reverse)

5'-ATAGG CGC GCC TTA AAG TGA AAC AAA ATT ATT TCT CTT -3'

(AscI: GG CGC GCC; Stop: TTA; pSL-3xGFP-sequence: AAG TGA AAC AAA ATT ATT TCT CTT)

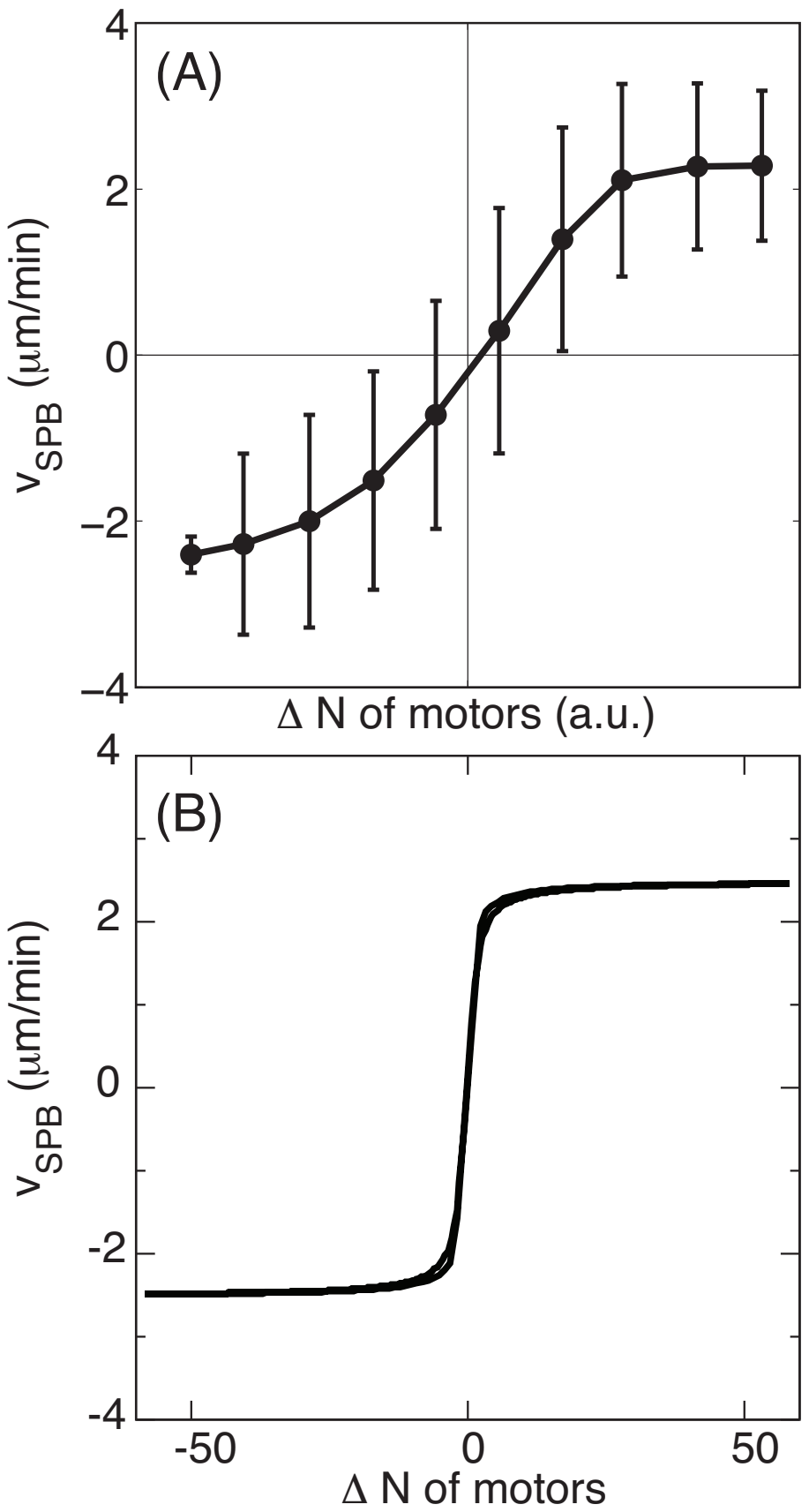

Figure S11: Experimental measurements (A) and numerical calculation (B) of the velocity of the SPB, v_{SPB}, as a function of the difference in the number of motors, ΔN of motors, on either side of the SPB. The number of motors in (A) is in arbitrary units, and was calculated from the dynein intensity. For details see Subsection II G 5. The points represent the mean ± s.d. The total number of data points is 3000, calculated from 13 cells. For the numerical calculation (B), the parameter values are as in Fig. 4G.

The 3xGFP sequence was subsequently subcloned into the AscI and PacI digested pFA6a-GFP-kanMX6 plasmid (kind gift from J. Bähler) by replacing the single GFP sequence with the triple GFP sequence to obtain pSV03. Oligonucleotides containing eighty basepaires of homologous flanking sequences of dhc1 were designed using a web based tool named PPPP [S16] available at `www.sanger.ac.uk/PostGenomics/S_pombe/software/` The following primer pair was used:

1. Forward-Primer (SV-forward-dhc1)
5'-ATGAAATTCCACCGGGCTGGTTGGACATACC
GGAAAACAGTAAGCGAAAGAGAACTGATATTT
ATTTTAGTATGTGCATTCGGATCCCCGGGTTAA
TTAA -3'

2. Reverse-Primer (SV-reverse-dhc1)
5'-AAAATTATACTAGGAGTTTTAATGGGAGGAC
AATAAAAGTTACGACAAAGTCTTTAGTAAACA
ACAAATAGTTTCAAGTTGAATTCGAGCTCGTT
TAAAC -3'

Using these primers and plasmid pSV03 as a template, DNA fragments of 4.2 kilobase length including the 3xGFP sequence and the kanamycin cassette were PCR amplified. Products from eight PCR reactions (40 μl each reaction) were pooled and purified using the QIAquick PCR Purification Kit for microcentrifuges (QIAGEN) to obtain 10 μl with more than 10 μg DNA fragments. The strain CRL152 (kind gift from Y. Hiraoka) was transformed with the PCR fragments using a chemical transformation method based on Lithium-acetate [S17], [S15] to obtain SV56 which was used as the parent strain for the subsequent strain construction of SV81 (see below).

2. *mCherry tagging of alpha2-tubulin (atb2) and subsequent integration into the ars1 locus of the strain SV56*

To generate a fission yeast strain expressing mCherry-atb2 together with endogenous levels of wild-type atb2, we first modified the GFP-fusion protein expression plasmid pDQ105 [S18] by replacing the GFP sequence with the mCherry sequence. For these purposes the ORF of mCherry was PCR amplified with the flanking restriction sites NdeI and BamHI from the pCS2-plasmid (kind gift from R. Tsien). The following primer pair was used:

1. Forward-Primer (SV-NdeI-mCherry-start)
NdeI start
5'-AAACAT ATG ATG
GTG AGC AAG GGC GAG GAG GAT -3'
mCherry sequence

2. Reverse-Primer (SV-BamHI-mCherry-end)
BamHI
5'-TTA GGA TCC
CTT GTA CAG CTC GTC CAT GCC GCC-3'
mCherry sequence

The mCherry sequence was inserted into the NdeI and BamHI digested pDQ105 plasmid by replacing the GFP with the mCherry sequence to obtain pSV01. To integrate amino-terminally tagged atb2 into the genome in addition to the wild-type atb2^{+} gene, pSV01 was digested with MluI. The MluI restriction site is located within the ars1 sequence of the pSV01 plasmid. The ars1 sequence is located in the genome on chromosome I. Approximately 1 μg plasmid DNA was used for the digestion with MluI and subsequently purified using the QIAquick PCR Purification Kit for microcentrifuges (QIAGEN) to obtain 10 μl, containing more than 1 μg DNA. The linearized plasmid containing homologous flanking sequences to the ars1 locus was transformed into the SV56 strain using a Lithium-acetate based transformation method [S17], [S15] followed by the

isolation of stable integrants which were found to exhibit uniform mCherry-atb2 expression by fluorescence microscopy (unpublished data). The obtained strain SV81 expressed endogenous levels of wild-type atb2 together with N-terminal mCherry-tagged atb2 from the ars1 locus under the control of the *nmt1*-promotor.

The strains SV28 and SV31 were obtained by crossing the strain P3-GFP-atb2 (kind gift from T. Toda) with KGY2679 (kind gift from K. Gould) using standard genetic methods, as previously described [S19].

E. Time-lapse fluorescence microscopy

Live cell imaging was performed on either of the following four microscopes:

(1) Yokogawa scan head CSU10 spinning disk system connected to an Olympus IX71 inverted microscope with an Andor Ixon (DU-897E-CS0-#BV) 512x512 EMCCD camera and an Argon, Krypton mixed gas laser with 488 nm and 568 nm laser lines (Innova). For simultaneous GFP and mCherry excitation, the 488 nm and the 568 nm laser lines, an UPLanSApo 100x/1.40 Oil UIS2 objective (Olympus), and a dichroic beamsplitter Di01-T405/488/568/647-13x15x0.5 (Semrock) were used. The xy-pixel size was 167 nm, the z-distance between optical sections 500 nm, and the time between stacks 8 s.

(2) Olympus FV-1000 laser scanning confocal system connected to an Olympus IX-81 inverted microscope with a 488 nm multi-line Argon laser (Melles Griot). For GFP excitation, the 488 nm laser line, a primary dichroic mirror DM 405/488, an UPLanSApo 60x/1.35 Oil LSM UIS2 objective (Olympus), and for emission a DM 500-600 nm were used. The xy-pixel size was 40 or 100 nm, the z-distance between optical sections 500 nm, and the time between stacks 8 s.

(3) Custom-built 2-Photon setup [S20] based on a Coherent Ti:Sa femtosecond laser (Chameleon-XR), an Hamamatsu photomultiplier (H9305-03), a Stanford Research low-noise current to voltage amplifier (SR-570), a couple of GSI-Lumonics galvanometer mirrors (VM1000) controlled by two mini-SAX boards, and a Zeiss objective (Plan-Apochromat 100x/1.40 Oil). The laser was tuned at 895 nm for GFP excitation, with an average power of 2 mW on the sample. A Chroma primary dichroic (650dcspxr), IR-blocking filter (HC 680/SP) and emission filter (ET525/50m) were also used in the detection path. Data acquisition was performed using a National Instruments AD/DA card (PCI-6295 M series DAQ board) controlled by a custom-written software developed in Labview (Version 8.2) running on a Dell computer (Intel Pentium D, 3.00 GHz, 1.00 GB of RAM) using Microsoft Windows XP. The xy-pixel size was 100 nm, the z-distance between optical sections 500 nm, and the time between stacks 7-15 s.

(4) Perkin Elmer UltraView VoX spinning disk system with a Yokogawa CSU X-1 scan head, fast emission filter wheel and an automatic dichroic changer connected to an Axiovert 200M inverted microscope with a Hamamatsu cooled 1000x1000 EMCCD 9100-02 camera and a laser combiner unit with 6 laser lines: 405, 445, 488, 514, 561 and 640. For sequential GFP and mCherry excitation, the 488 and the 561 laser lines, and a CFI Plan Apo TIRF 100x/1.45 oil objective (Nikon) were used. Data acquisition was controlled by Volocity software. The xy-pixel size was 80 nm, the z-distance between optical sections 500 nm, and the time between stacks 8 s.

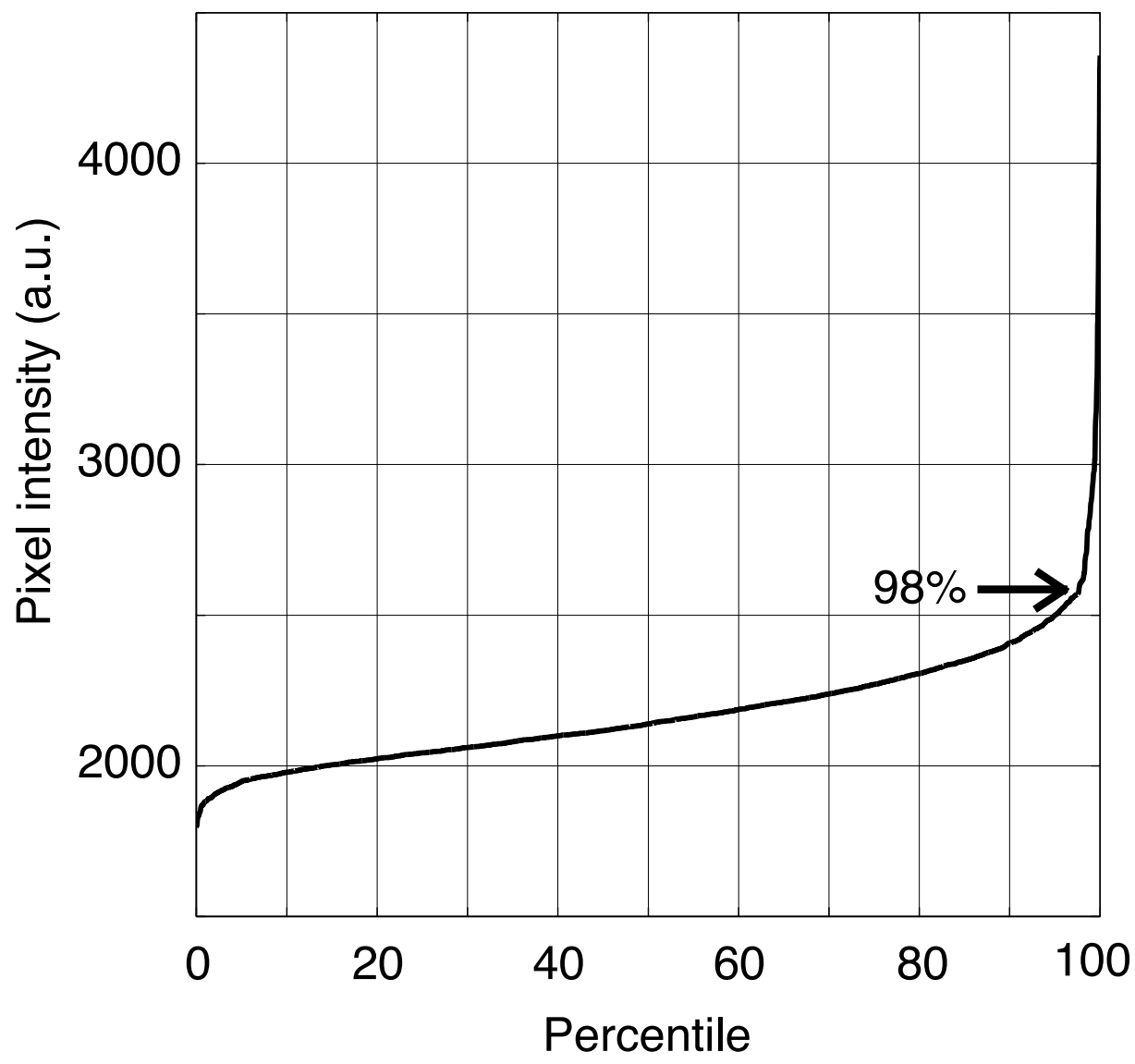

Figure S12: Plot of sorted intensity values. We chose the 98th percentile as a threshold for the quantification of dynein.

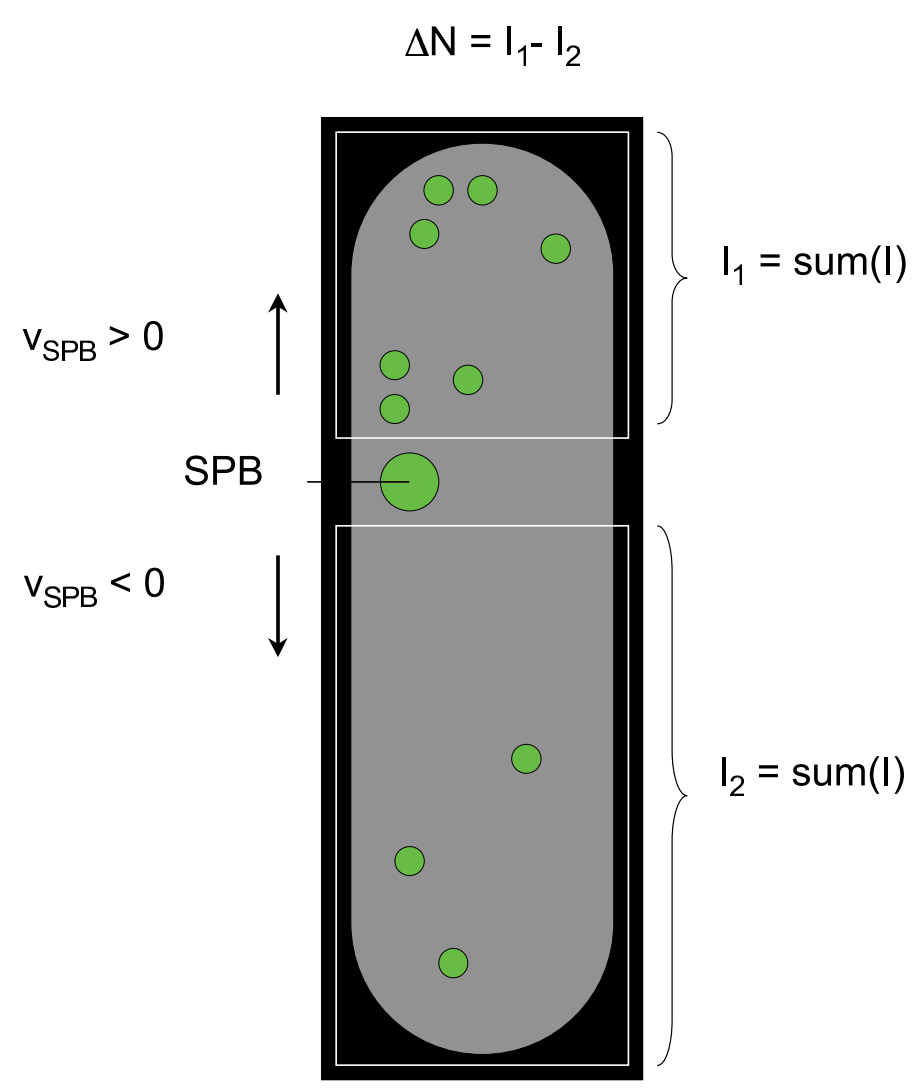

Figure S13: Schematic representation of the algorithm for dynein quantification.

F. Laser ablation

Laser ablation was performed on microscopes (2) and (3):

(2) Olympus FV-1000 laser scanning confocal system
Laser cutting was performed using tornado mode of the SIM scanner of FV-1000 and a PDL 800-B picosecond pulsed diode laser with an LDH-P-C-405B laser head (PicoQuant) emitting 70 picosecond pulses at 40 MHz. The average power at the sample was 0.4 mW. Laser power was measured before the objective using a power-meter (Thorlabs). The measured power was later corrected for the objective's transmission at 405 nm (80%). The laser exposure time was 2 seconds and controlled by an external time controller (PicoQuant). The system was driven by FluoView Application Software version 1.6a (Olympus) run on a PC -Windows XP.

(3) Custom-built 2-Photon setup [S20]
Laser cutting, performed while continuously scanning a stack, was achieved by focusing the laser over a user-defined point and increasing the average power to 70 mW on the sample. Exposure time, controlled by the computer, was 150 ms.

G. Image analysis

1. *Spindle pole body tracking in the strain SV28xSV31 expressing Sid4-GFP and GFP-atb2 (Figs. 1 and 6)*

The SPB position was tracked manually from the maximum-intensity projections of the z-stacks in Image J (National Institutes of Health, USA). The accuracy of the manual tracking was validated by automatic tracking using Spot tracker 2D plug-in by Daniel Sage (Biomedical Imaging Group, 2005 Swiss Federal Institute of Technology Lausanne (EPFL)) in Image J.

2. *Color merge of images from the strain SV81 expressing Dhc1-3GFP and mCherry-tubulin (Figs. 2 and 3)*

The color-merge images shown in Figs. 2 and 3 were obtained by merging the maximum-intensity projections of the green and the red channel corresponding to the Dhc1-3GFP and mCherry-tubulin signal, respectively. The maximum-intensity projections were calculated in Image J for each channel, and then merged using the Image J Colour merge plug-in.

3. *Kymograph (Fig. 2E)*

Each column in the kymograph represents the maximum projection along the short cell axis of the image showing the sum-intensity projections of the green (dynein) channel thresholded at the 98th percentile of the intensity values.

4. *Line profiles (Fig. 3 and S8C, D)*

The line profiles in Fig. 3 were calculated by using Image J. In Fig. S8C, D a stack of 10 sections was acquired at the Olympus confocal microscope with a pixel size of 40 nm and a scanning speed of 2 μs/pixel. The maximum-intensity projections were calculated in Image J for the green and the red channel separately, and filtered with a median filter on 5x5 pixel neighborhoods, using the medfilt2 function in Matlab (Mathworks). Line profiles in Fig. S8D were calculated using the Matlab improfile function. For clarity, a neighboring cell was removed from the image in Fig. S8C.

5. *Analysis of dynein dynamics and SPB tracking in the strain SV81 expressing Dhc1-3GFP and mCherry-tubulin (Fig. 5)*

Dynein intensity analysis was performed using programs written in Matlab. Sum-intensity projections of the green (dynein) channel were calculated and thresholded at the 98th percentile of the intensity values. This threshold was chosen because plots of sorted intensity values showed that the signal was in the 2% pixels with the highest intensities (Fig. S12). The SPB position (x_{SPB}, Fig. 5D) was found automatically by a tracking program that finds the center-of-mass of the brightest region in the image. The resulting tracks were checked visually. Since the signal in the thresholded images roughly corresponded to dynein on the microtubules, the total amount of dynein on the upper microtubules (Fig. 5F, red) was calculated as the sum of intensities of all pixels situated more than 4 pixels above the SPB position (I1 in Fig. S13). The amount of dynein on the lower microtubules (Fig. 5F, blue) was the sum of intensities of pixels situated more than 4 pixels below the SPB position (I2 in Fig. S13).

H. Supplemental Movie Legends

1. *Supplemental Movie 1: Spindle pole body (SPB) movement via microtubules*

SPB movement in a zygote during meiotic prophase expressing Sid4-GFP (SPB marker) and GFP-alpha2-tubulin. The zygote was obtained by crossing of strains SV28 and SV31. Images were acquired at 4 second intervals. The video is displayed at 10 frames per second (fps). Total time: 14 min. (avi; 0.67 MB)

2. *Supplemental Movie 2: Laser ablation of the leading microtubules affects the SPB movement*

A meiotic prophase zygote expressing Sid4-GFP (SPB marker) and GFP-alpha2-tubulin where the ablation of

the front microtubules leads to a change in the direction of the SPB motion. The red arrowhead marks the ablation position. The movie pauses for 2 seconds at the ablation time. The zygote was obtained by crossing of strains SV28 and SV31. Images were acquired at 7 second intervals. The video is displayed at 5 fps. Total time: 10 min 15 sec. Corresponds to Fig. 1B. (avi; 0.86 MB)

3. Supplemental Movie 3: Laser ablation of the trailing microtubules does not affect the SPB movement

A meiotic prophase zygote expressing Sid4-GFP (SPB marker) and GFP-alpha2-tubulin where the ablation of the trailing microtubules does not influence the SPB movement. The red arrowhead marks the ablation position. The movie pauses for 2 seconds at the ablation time. The zygote was obtained by crossing of strains SV28 and SV31. Images were acquired at 12.5 second intervals. The video is displayed at 5 fps. Total time: 14 min 35 sec. Corresponds to Fig. 1C. (avi; 0.51 MB)

4. Supplemental Movie 4: Laser ablation of the tip of the leading microtubules does not affect the SPB movement

A meiotic prophase zygote expressing Sid4-GFP (SPB marker) and GFP-alpha2-tubulin where the ablation of the tip of the leading microtubules does not influence the SPB movement. The red arrowhead marks the ablation position. The movie pauses for 2 seconds at the ablation time. The zygote was obtained by crossing of strains SV28 and SV31. Images were acquired at 10.6 second intervals. The video is displayed at 5 fps. Total time: 9 min. Corresponds to Fig. 1D. (avi; 0.58 MB)

5. Supplemental Movie 5: Dynein signal is stronger on the leading than on the trailing microtubules

A meiotic prophase zygote expressing Dhc1-3GFP and mCherry-alpha2-tubulin (strain SV81). Note that the leading microtubules show a strong dynein signal while the dynein signal along the trailing microtubules is weak. Images were acquired at 8 second intervals. The video is displayed at 15 fps. Total time: 64 min 56 sec. Corresponds to Figs. 2A and S6. (avi; 4.3 MB)

6. Supplemental Movie 6: Dynein is distributed in a spotted pattern along the leading microtubules

A meiotic prophase zygote expressing Dhc1-3GFP and mCherry-alpha2-tubulin (strain SV81). Note that dynein is distributed in a spotted pattern along the leading microtubules. The spotted pattern is stationary with respect to the cortex. Images were acquired at 8 second intervals. The video is displayed at 15 fps. Total time: 17 min 28 sec. Corresponds to Figs. 2B and S9. (avi; 1.1 MB)

7. Supplemental Movie 7: Dynein remains on the end of depolymerizing microtubules

A meiotic prophase zygote expressing Dhc1-3GFP and mCherry-alpha2-tubulin (strain SV81). Note that when the microtubule detaches from the cortex, dynein remains on the plus-end during microtubule depolymerization. Images were acquired at 8 second intervals. The video is displayed at 10 fps. Total time: 1 min 4 sec. Corresponds to Fig. 2C. (avi; 48 KB)

[S1] A. Gennerich, A. P. Carter, S. L. Reck-Peterson, and R. D. Vale, "Force-induced bidirectional stepping of cytoplasmic dynein," *Cell*, vol. 131, pp. 952–965, Nov 2007.

[S2] S. Toba, T. M. Watanabe, L. Yamaguchi-Okimoto, Y. Y. Toyoshima, and H. Higuchi, "Overlapping hand-over-hand mechanism of single molecular motility of cytoplasmic dynein," *Proc. Natl. Acad. Sci. USA*, vol. 103, pp. 5741–5745, April 2006.

[S3] R. Mallik, B. C. Carter, S. A. Lex, S. J. King, and S. P. Gross, "Cytoplasmic dynein functions as a gear in response to load.," *Nature*, vol. 427, no. 6975, pp. 649–52, 2004.

[S4] S. L. Reck-Peterson, A. Yildiz, A. P. Carter, A. Gennerich, N. Zhang, and R. D. Vale, "Single-molecule analysis of dynein processivity and stepping behavior.," *Cell*, vol. 126, no. 2, pp. 335–48, 2006.

[S5] S. W. Grill, K. Kruse, and F. Jülicher, "Theory of mitotic spindle oscillations.," *Phys. Rev. Lett.*, vol. 94, March 2005.

[S6] C. Kozlowski, M. Srayko, and F. Nedelec, "Cortical microtubule contacts position the spindle in c. elegans embryos.," *Cell*, vol. 129, pp. 499–510, May 2007.

[S7] W.-L. Lee, J. R. Oberle, and J. A. Cooper, "The role of the lissencephaly protein pac1 during nuclear migration in budding yeast.," *J Cell Biol*, vol. 160, no. 3, pp. 355–364, 2003 Feb 3.

[S8] K. E. Busch and D. Brunner, "The microtubule plus end-tracking proteins mal3p and tip1p cooperate for cell-end targeting of interphase microtubules.," *Curr Biol*, vol. 14, no. 7, pp. 548–559, 2004 Apr 6.

[S9] J. L. Hoog, C. Schwartz, A. T. Noon, E. T. O'Toole, D. N. Mastronarde, J. R. McIntosh, and C. Antony, "Organization of interphase microtubules in fission yeast analyzed by electron tomography.," *Dev Cell*, vol. 12, no. 3, pp. 349–361, 2007 Mar.

[S10] M. J. Sagolla, S. Uzawa, and W. Z. Cande, "Individual microtubule dynamics contribute to the function of mitotic and cytoplasmic arrays in fission yeast.," *J Cell Sci*, vol. 116, no. Pt 24, pp. 4891–4903, 2003 Dec 15.

[S11] J. Mata, R. Lyne, G. Burns, and J. Bahler, "The transcriptional program of meiosis and sporulation in fission yeast.," *Nat Genet*, vol. 32, no. 1, pp. 143–147, 2002 Sep.

[S12] A. Yamamoto, C. Tsutsumi, H. Kojima, K. Oiwa, and Y. Hiraoka, "Dynamic behavior of microtubules during dynein-dependent nuclear migrations of meiotic prophase in fission yeast.," *Mol Biol Cell*, vol. 12, no. 12, pp. 3933–3946, 2001 Dec.

[S13] J. H. Lenz, I. Schuchardt, A. Straube, and G. Steinberg, "A dynein loading zone for retrograde endosome motility at microtubule plus-ends.," *EMBO J*, vol. 25, no. 11, pp. 2275–2286, 2006 Jun 7.

[S14] J. Zhang, S. Li, R. Fischer, and X. Xiang, "Accumulation of cytoplasmic dynein and dynactin at microtubule plus ends in aspergillus nidulans is kinesin dependent.," *Mol Biol Cell*, vol. 14, no. 4, pp. 1479–1488, 2003 Apr.

[S15] J. Bähler, J.-Q. Wu, M. S. Longtine, N. G. Shah, A. M. III, A. B. Steever, A. Wach, P. Philippsen, and J. R. Pringle, "Heterologous modules for efficient and versatile pcr-based gene targeting in Schizosaccharomyces pombe," *Yeast*, vol. 14, no. 10, pp. 943–951, 1998.

[S16] C. J. Penkett, Z. E. Birtle, and J. Bähler, "Simplified primer design for pcr-based gene targeting and microarray primer database: two web tools for fission yeast," *Yeast*, vol. 23, no. 13, pp. 921–928, 2006.

[S17] S. L. Forsburg and N. Rhind, "Basic methods for fission yeast.," *Yeast*, vol. 23, no. 3, pp. 173–183, 2006.

[S18] D.-Q. Ding, Y. Chikashige, T. Haraguchi, and Y. Hiraoka, "Oscillatory nuclear movement in fission yeast meiotic prophase is driven by astral microtubules, as revealed by continuous observation of chromosomes and microtubules in living cells," *J Cell Sci*, vol. 111, no. 6, pp. 701–712, 1998.

[S19] S. Moreno, A. Klar, and P. Nurse, "Molecular genetic analysis of fission yeast Schizosaccharomyces pombe.," *Methods Enzymol*, vol. 194, 1991.

[S20] N. Maghelli and I. Tolic-Nørrelykke, "Versatile laser-based cell manipulator," *J Biophoton*, vol. 1, no. 4, pp. 299–309, 2008.
